# Supplementary material for: Histopathological profile of cervical biopsies in northern Malawi: a retrospective cross-sectional study
Source: BMJ Open. 2022 Mar 11;12(3):e048283. doi: 10.1136/bmjopen-2020-048283 (PMC8919446; doi:10.1136/bmjopen-2020-048283)
Supplement: Author's manuscript [file bmjopen-2020-048283.draft_revisions.pdf]

BMJ Open

BMJ Open is committed to open peer review. As part of this commitment we make the peer review history of every article we publish publicly available.

When an article is published we post the peer reviewers' comments and the authors' responses online. We also post the versions of the paper that were used during peer review. These are the versions that the peer review comments apply to.

The versions of the paper that follow are the versions that were submitted during the peer review process. They are not the versions of record or the final published versions. They should not be cited or distributed as the published version of this manuscript.

BMJ Open is an open access journal and the full, final, typeset and author-corrected version of record of the manuscript is available on our site with no access controls, subscription charges or pay-per-view fees (http://bmjopen.bmj.com).

If you have any questions on BMJ Open's open peer review process please email
info.bmjopen@bmj.com

BMJ Open

Histopathological Profile of Cervical Biopsies in Northern Malawi: A Retrospective Study

Journal:	*BMJ Open*
Manuscript ID	bmjopen-2020-048283
Article Type:	Original research
Date Submitted by the Author:	03-Jan-2021
Complete List of Authors:	Kaseka, Paul; Mzuzu Central Hospital,<br>Kayira, Alfred; Mzuzu Central Hospital<br>Chimbatata, Chikondi; Mzuzu Central Hospital<br>Chisale, Master; Mzuzu University, Biological Sciences; Mzuzu University<br>Kamudumuli, Pocha; University of Maryland and Global Initiative Company<br>Wu, Tsung-Shu; Ping Tung Christian Hospital<br>Mbakaya, Balwani; University of Livingstonia<br>Sinyiza, Frank; Mzuzu Central Hospital
Keywords:	Pathology < TROPICAL MEDICINE, ONCOLOGY, OBSTETRICS

SCHOLARONE™
Manuscripts

I, the Submitting Author has the right to grant and does grant on behalf of all authors of the Work (as defined in the below author licence), an exclusive licence and/or a non-exclusive licence for contributions from authors who are: i) UK Crown employees; ii) where BMJ has agreed a CC-BY licence shall apply, and/or iii) in accordance with the terms applicable for US Federal Government officers or employees acting as part of their official duties; on a worldwide, perpetual, irrevocable, royalty-free basis to BMJ Publishing Group Ltd ("BMJ") its licensees and where the relevant Journal is co-owned by BMJ to the co-owners of the Journal, to publish the Work in this journal and any other BMJ products and to exploit all rights, as set out in our licence.

The Submitting Author accepts and understands that any supply made under these terms is made by BMJ to the Submitting Author unless you are acting as an employee on behalf of your employer or a postgraduate student of an affiliated institution which is paying any applicable article publishing charge ("APC") for Open Access articles. Where the Submitting Author wishes to make the Work available on an Open Access basis (and intends to pay the relevant APC), the terms of reuse of such Open Access shall be governed by a Creative Commons licence – details of these licences and which Creative Commons licence will apply to this Work are set out in our licence referred to above.

Other than as permitted in any relevant BMJ Author's Self Archiving Policies, I confirm this Work has not been accepted for publication elsewhere, is not being considered for publication elsewhere and does not duplicate material already published. I confirm all authors consent to publication of this Work and authorise the granting of this licence.

For peer review only

Histopathological Profile of Cervical Biopsies in Northern Malawi: A Retrospective Study

Paul Uchizi Kaseka[1*], Alfred Kayira[1], Chikondi Sharon Chimbatata[1], Master Chisale[1,2,3,] Pocha Kamudumuli[4], Tsung-Shu Joseph Wu[2,5], BalwaniChingatichifwe Mbakaya,[6,7]Frank Watson Sinyiza[1]

[1]Mzuzu Central Hospital, Mzuzu City, Malawi

[2]Luke International, Mzuzu City, Malawi

[3]Mzuzu University, Faculty of Sciences, Technology and Innovations, Biological Sciences, Mzuzu City, Malawi

[4]University of Maryland Global Initiative Corporation, Baltimore, Lilongwe City, Malawi

[5]Pingtung Christian Hospital, Taiwan

[6]University of Livingstonia, Department of Public Health, Malawi

[7]Mzuzu University, Faculty of Health Sciences, Mzuzu City, Malawi

*Corresponding author: kasekapaul2016@gmail.com

ABSTRACT

Objectives: This study was carried out to determine the histopathological profile of cervical biopsies in a public tertiary hospital in Mzuzu, northern region of Malawi.

Setting: A public tertiary hospital in Mzuzu, northern region of Malawi

Participants: This was a retrospective study of all cervical biopsy specimen reports received in a public tertiary hospital in northern Malawi over a period of 5 years from July 2013-June 2018. Eleven reports which had missing demographic and clinical data or had inconclusive results were excluded. Demographic, clinical and histopathological data was obtained from original histology reports.

Results: A total of 500 cervical biopsy reports were reviewed during the study period. The mean age of the patients was 41.99±12.5. Age ranged from 15 to 80 years. Cervicitis accounted for 46.0% (n=162) of the total nonmalignant lesions seen, followed by cervical intraepithelial neoplasm (CIN), at 24.4% (n=86) and endocervical polyp, at 20.5% (n=72). Squamous cell carcinoma (SCC) accounted for 15.6% (n=78) of the total

For peer review only

cervical biopsies studied and 85.7% of all total malignant lesions. All malignant tumours had HIV.

Conclusion: Our study shows that cervicitis and squamous cell carcinoma were most common among nonmalignant and malignant cervical biopsies respectively. Since the frequency of cervical cancer is high, there is need to have well detailed national policies to be put in place to increase detection of pre-invasive lesions in order to reduce the prevalence of cervical cancer.

Key words: Cervical biopsies, cervical cancer, cervical intraepithelial neoplasm, cervicitis, malignant

Strengths and limitation of this study

Strengths

This paper has shown

- The need for well detailed national policies to be put in place to increase detection of pre-invasive lesions, which in turn will decrease the frequency of cervical cancer in the country.
- The importance of intensifying cervical cancer screening programmes among women and provision of long term ART to the HIV infected which may offer an opportunity for appropriate interventions to reduce morbidity, mortality and reduce complications among these women.

Limitations

- This study used available programme health facility data and histopathological reports on cervical cancer which has its own limitations, such as incompleteness and bias in the sense that information is obtained only from people who came to the facility and underwent biopsy, leaving out those that did not seek medical care and or were not biopsied and therefore cannot be generalized to the general population.
- The study is a single-hospital-based review and as such inadequate to draw

For peer review only - http://bmjopen.bmj.com/site/about/guidelines.xhtml

conclusions, but it does shed some light on pathological pattern of cervical cancer in Malawi.

BACKGROUND

Cervical cancer, after breast cancer, is the second most common cancer in women aged 15 to 44 years and it is the third leading cause of cancer in females worldwide(1). According to the International Agency for Research on Cancer (IARC) estimates, there are 570,000 new cases of cervical cancer annually, resulting into more than 311,000 deaths in 2018 globally (2). Most of the global burden lies in less developed countries, with sub-Saharan Africa (SSA) having the largest age-standardized incidence and mortality rates. Malawi has the highest cervical cancer incidence and mortality in the world with age-standardized rate (ASR) of 75.9 and 49.8 per 100,000 population respectively(3). World Health Organization (WHO) estimates suggest that every year there are at least 3,684 new cases of cervical cancer in Malawi and over 2,314 die from the disease(3).The exact number of cervical cancer morbidity and mortality among Malawian women, is not clear. This could partly be due to unrecorded or underreported cases because of the pathological based cancer registry not being maintained, and also lack of a national system of death certification(4). However, the 2010 National population-based cancer registry indicates that among females, cancer of the cervix was the commonest, accounting for 45.4% of all cases followed by Kaposis sarcoma (21.1%), cancer of the oesophagus (8.2%), breast cancer (4.6%) and non-Hodgkin lymphoma (4.1%)(5).

The standard method for diagnosis of cervical precancerous lesions is histopathological examination of tissue obtained through biopsy guided by colposcopy. When abnormalities are identified, cervical biopsy confirms the diagnosis of cancer(6).This study was carried out to determine the histopathological profile of cervical biopsies in a public tertiary hospital in Mzuzu, northern region of Malawi. The specific objectives were to determine the prevalence of both precancerous and cancerous cervical lesions; characterization of precancerous and cancerous lesions and risk factors of cervical

For peer review only - http://bmjopen.bmj.com/site/about/guidelines.xhtml

cancer. Understanding the histological pattern of cervical cancer could guide development of focused preventive, care and treatment guidelines to address shortfalls in the care that is provided to cervical cancer patients in Malawi. Ultimately, this could contribute to the global target of 25% reduction of premature mortality from non communicable diseases (NCDs) by the year 2025.

MATERIALS AND METHODS

Design, setting and population

This was a retrospective study of all cervical biopsies reports received from Kamuzu Central Hospital/University of North Carolina (KCH/UNC) pathology laboratory in Lilongwe for Mzuzu Central Hospital (MCH), the only public tertiary hospital in the northern region of Malawi. This hospital does not have a functional pathology laboratory and relies on the KCH/UNC pathology laboratory for its services. The hospital is located in the northern part of Malawi catering for a population of about 2,289,780 million people (7) and serving 5 government District Hospitals, 3 Christian Health Association of Malawi (CHAM) hospitals and several private hospitals and clinics**.**

In this study a total of 500 cervical cancer pathology reports were analyzed over a period of 5 years (June 2013 to July 2018). Eleven reports which had missing demographic and clinical data or had inconclusive results were excluded. Data extracted included age, year, anatomic site, nature of specimen, clinical diagnosis, histopathological diagnosis, HIV status and whether the specimen was nonmalignant or malignant.

Patient and public involvement

No patient involved

Data analysis

For peer review only - http://bmjopen.bmj.com/site/about/guidelines.xhtml

Data were entered in Microsoft excel 2016, validated and cleaned before importing into Stata, version 13.0 (Stata Corp. LP, College Station, TX, United States of America) for analysis. Descriptive analyses were performed to summarize patients' sociodemographic and clinical characteristics. Chi Square (or Fisher's exact) test was used to look for significant associations between predictor and outcome variables at 95%significance level. A multiple simple logistic regression was used to quantify the association between predictor variables and outcome variables.

Ethical clearance

This study was approved by National Health Science Research Committee (NHSRC) as part of the main study "Pathological profile of malignancies in northern Malawi-A retrospective study at Mzuzu Central Hospital" number 19/05/2316. Both the MCH Research and Publication Committee and the MCH Laboratory department consents were obtained for the study. The need for informed consent was exempted from the institutional review board due to the nature of the study, which involved a retrospective analysis of routinely collected data.

RESULTS

Within the 5-year period of the study (July 2013 to June 2018), a total of 500 biopsy reports were received from KCH/ UNC Pathology laboratory. The mean age of patients included in this study was 41.99 ± 12.5. The age range was 15-80 years. Most of the cervical biopsies were from patients in the 31-40 age group (34.8% n=174). Twelve percent of the cervical biopsies were from HIV positive patients and 51% of the results had unknown HIV result (Table 1).

Table 1. Demographic profile of cervical biopsies

**Parameter**		**Biopsies analysed**	
		**Frequency**	**Percentage (%)**
Age (years)	20 and below	3	0.6
	21-30	57	11.4

For peer review only - http://bmjopen.bmj.com/site/about/guidelines.xhtml

	31-40	174	34.8
	41-50	132	26.4
	51-60	84	16.8
	61-70	29	5.8
	Above 70	7	1.4
	Missing	14	2.8
HIV status	Positive	61	12.2
	Negative	184	36.8
	Unknown (not documented)	255	51.0

Of all the cervical biopsies studied, 91 (18.2%) were malignant. Squamous cell carcinoma (SCC) accounted for 85.7% of all malignant lesions (Table 2). Cervicitis accounted for 46.0% (n=162) of the total nonmalignant lesions seen. Ten percent of cervicitis cases and 30.2% of CIN cases had HPV respectively (Table 2).

Table 2. Histopathological diagnosis of cervical biopsies

Parameter	Frequency	Within group percentage(%)	Percentage(%)total
**Normal cervical tissue**	**46**		**9.2**
**Nonmalignant lesions**	**352**		**70.4**
Cervicitis*	162	46.0	32.4
Cervical intraepithelial neoplasm (CIN)*	86	24.4	17.2
Endocervical polyp	72	20.5	14.4
Carcinoma in situ	4	1.1	0.8
Narborthian cyst	4	1.1	0.8
Schistosomiasis	5	1.4	1.0
Condloma (warts)	11	3.1	2.2

For peer review only - http://bmjopen.bmj.com/site/about/guidelines.xhtml

Others	8	2.3	1.6
**Malignant**	**91**		**18.2**
Squamous cell carcinorma	78	85.7	15.6
Adenocarcinorma	8	8.8	1.6
Poorly differentiated	4	4.4	0.8
Carcino-sarcoma	1	1.1	0.2
**Inconclusive result**	**11**		**2.2**
**Total**	**500**		**100**

*Cervicitis with HPV= 10% (16/162)

*CIN with HPV= 30.2% (26/86)

*CIN 1 = 34.9% (30/86), CIN 2 = 22.1% (19/86), CIN 3 = 43.0% (37/86)

The chances of developing cervical cancer increased with advancing age, with the risk becoming more prominent after age 60 years. Women aged 61-70 years were 6.2 times (OR = 6.2, 95% CI: 2.1 - 18.4) more likely to develop cervical cancer than those in the 21-30 years' age category. The odds of getting cancer were higher in women living with HIV. Women with HIV were more than twice (OR = 2.3, 95% CI: 1.1 - 4.9) as likely to have cervical cancer as those who were HIV negative (Table 3).

For peer review only

Table 3: Association between cancer, age and HIV status

		Cancer (%)	Non cancer (%)	Unadjusted odds ratio (95% CI)†	Adjusted odds ratio (95% CI)†
Age (years)	20 and below	0 (0.0)	2 (100.0)		
	21 - 30 (reference)	7 (12.3)	50 (87.2)	-	
	31 - 40	25 (14.6)	146 (85.4)	1.2 (0.5 -3.0)	1.2 (0.5- 2.9)
	41 - 50	23 (17.7)	107 (82.3)	1.5 (0.6 - 3.8)	1.5 (0.6 - 3.8)
	51 - 60	18 (22.2)	63 (77.8)	2.0 (0.8 - 5.3)	2.2 (0.8 - 5.7)
	61 - 70	13 (44.8)	16 (55.2)	5.8 (2.0 - 17.0)	6.2 (2.1 - 18.4)
	Above 70	1 (20.0)	4 (80.0)	1.8 (0.2 - 18.3)	1.9 (0.2 - 19.9)
HIV status	Negative (reference)	26 (14.4)	155 (85.6)	-	
	Positive	15 (24.6)	46 (75.4)	1.9 (1.0 - 4.0)	2.3 (1.1 - 4.9)
	Unknown	50 (20.2)	197 (79.8)		

†Binary logistic regression

For peer review only

DISCUSSION

To the best of our knowledge, this is the first study conducted to determine the histopathological profile of cervical biopsies in a public tertiary hospital in Mzuzu, northern region of Malawi. This study found more benign conditions (70.4% n=352) than neoplastic malignant conditions (18.2% n=91). This is in contrast with other studies undertaken in developing countries which reported more neoplastic malignant conditions than benign conditions(8,9).

Our study found that cervicitis, an inflammatory disease comprising acute cervicitis and chronic non-specific cervicitis was the most common nonmalignant condition. It accounted for 46% of all the nonmalignant tumours and 32.4% of all cervical biopsies received in this study. This was consistent to a study conducted in Nigeria whose cervical biopsies included in their study was 37.5%(8). Most cases of cervicitis are often due to non-specific causes or infective agents(9). Cervicitis has been highly reported in previously studies in other countries(12,13).

In this study CIN, a premalignant lesion ranked second in nonmalignant tumours accounting for 24% of all nonmalignant tumours and 17.2% of all cervical biopsies with the prevalence of CIN I, CIN II, CIN III of 34.9%, 22.1% and 43% respectively. This finding is comparable to a study conducted in Nigeria which reported CIN constituting 15% of all cervical biopsies reviewed(10). In that study however, there was a reduction in the prevalence of CIN from low grade CIN to high grade CIN(10). In our study, the high grade CIN (CIN III) was the most common. This finding indicates that invasive cervical cancer progresses from advanced stages of precancerous lesions. This shows that there is need to create community awareness and strengthen early cervical cancer screening for Malawi to have better outcomes.

In the current study 10% (n=16) of all the cervicitis cases and 30.2% (n=26) of all the CIN biopsies had HPV. This is comparable to a study conducted in United States of America (USA) which reported the overall HPV prevalence of 26.8% among US females aged 14 to 59 years (n=1921)(11). This finding suggests that there is high rate of HPV infections, the causative agent of CIN in the younger age group(10). Unfortunately, the

For peer review only - http://bmjopen.bmj.com/site/about/guidelines.xhtml

data on population-based, age specific prevalence of HPV are not available in Malawi. However, WHO estimates that the overall HPV prevalence in Malawi is about 34%(12).

The rate of malignant lesions (tumours) in our study (18.2%) is comparable to 16.2% and 12.2% in studies conducted in Benin City(13), and in Enugu, in Nigeria(14) respectively. Studies done in South Africa, Saudi Arabia, India and United States, with malignant lesions at 2.42%, 4.95%, 5.5% and 5.0% respectively, are all at variance with the current study which explains that early cervical cancer screening helps to reduce the prevalence of cervical cancer(15–18).

Among the malignant tumours, SCC was the most common histological type of cervical cancer in our study. SCC accounted for 85.7% of malignant lesions cervical cancer and was also the most common diagnosis, at 15.6% of all cervical biopsies in this study. This is consistent with findings from other studies conducted in Nepal and in Pakistan which reported 84.2%, 92.56%, and 73.5% respectively for squamous cell carcinoma out of all malignant lesions reviewed(19–21). As Faduyile et al. observed the high rate of SCC in Malawi and Africa could reflect the low uptake of VIA and Pap smear test which are capable of identifying dysplastic conditions before transformation to malignancy(11). This shows that there is need for Malawi to have well organized cervical cancer screening and Pap smear test to reduce the prevalence of SCC(2).

In our study, adenocarcinorma and poorly differentiated carcinorma were 8.8% and 4.4% respectively of the total malignant diagnosis. The prevalence of adenocarcinoma and poorly differentiated carcinoma observed in this study is in contrast to other studies done in Nigeria where one study found 5.8% and 2.0% of the total malignant lesions diagnosis and another found 6.0% and 1.0% respectively(10,22). The high prevalence found in this study confirms a previous study findings by Chanza et al.(23). Chanza et al. reported that Malawian women delay to seek medical attention due to limited knowledge on symptoms and signs, limited financial resources, limited accessibility and unavailability of cancer screening facilities hence late diagnosis. These results show that early cervical cancer screening, increased awareness, better health care facilities, accessibility, improved histopathological confirmatory diagnosis and early treatment by surgeons, may reduce the cervical cancer burden in Malawi.

For peer review only - http://bmjopen.bmj.com/site/about/guidelines.xhtml

In the current study it has been observed that the diagnosis of cervical cancer was significantly associated with the age of the patients. The chances of developing cervical cancer increased with advancing age, with the risk becoming more prominent after age 60 years. Women aged 61-70 years were 6.2 times (OR = 6.2, 95% CI: 2.1 - 18.4) more likely to develop cervical cancer than those in the 21-30 years age category (Table 3). It is important to note that high quality screening programs are important to prevent cervical cancer among unvaccinated older women(24). None of the women in our study had received any HPV vaccine in their lifetime as the first round of the mass HPV vaccine in this country was administered in January 2019 mainly in schools, targeting 9-13 year old girls who had not yet become sexually active and the second round was given in January 2020. In Malawi, the integration of HPV vaccine programs with adequate screening programs in older women (aged 30-49 years) has the potential to reduce the burden of cervical cancer.

Malawi's HIV prevalence is one of the highest in the world, with 10.6% of adult population (aged 15-64) living with HIV (25). With such a high HIV prevalence in Malawi, there is an increased risk of AIDS-defining cancers including cervical cancer. In this study the probability of getting cancer were higher in women living with HIV. Women with HIV were more than twice (OR = 2.3, 95% CI: 1.1 - 4.9)as likely to have cervical cancer as those who were HIV negative (Table 3).This could be attributed to the reason that HIV-infected women are more likely than HIV-uninfected women to have incident and persistent HPV cervical infections(26). A twelve monthly cervical cancer screening, increased availability of dolutegravir (DTG) based antiretroviral treatment (ART) to HIV infected women now being provided in the country, routine viral load checks (at 6 months, 12 months and every 12 months since initiation) and timely switch to second line or third line regimens to ensure viral load suppression will eventually have an impact on benign lesions progressing to invasive cervical cancer(27). However, in this study almost half (52.74%, n=48) of all the women diagnosed with cervical cancer had an unknown HIV status. This calls for scaling up of HIV testing in cancer screening settings for early diagnosis and ART referral and further research is warranted on barriers among HIV-infected women to seeking cancer screening services despite already being in the health care system.

For peer review only - http://bmjopen.bmj.com/site/about/guidelines.xhtml

LIMITATIONS

This study used available programme health facility data and histopathological reports on cervical cancer. The use of health facility data has its own limitations, such as incompleteness and bias in the sense that information is obtained only from people who came to the facility and underwent biopsy, leaving out those that did not seek medical care and or were not biopsied and therefore cannot be generalized to the general population.

The other limitation of this study is that it is a single-hospital-based review and as such inadequate to draw conclusions, but it does shed some light on pathological pattern of cervical cancer in Malawi.

Finally, this is a retrospective study so we could not be able to extract details for example in cases where tumours were diagnosed by screening or symptoms, presence or absence of the patient co-morbidities. Nevertheless, the comprehensive histopathological pattern of cervical cancer demonstrated by this study provides evidence that could be used to inform policies, strategies and intervention for prevention of cancer in Malawi.

CONCLUSION

The SCC was the commonest malignant condition and cervicitis and CIN were the most common non malignant conditions in all the women studied. Since the frequency of cervical cancer is high, there is need for well detailed national policies to be put in place to increase detection of pre-invasive lesions, which in turn will decrease the frequency of cervical cancer in the country. The presence of chronic non-specific cervicitis in women of reproductive age is infective in origin with its attending sequelae. Intensifying screening programmes among women and provision of long term ART to the HIV infected may offer an opportunity for appropriate interventions to reduce morbidity, mortality and reduce complications among these women.

For peer review only - http://bmjopen.bmj.com/site/about/guidelines.xhtml

REFERENCES

1. Bruni L, Albero G, Serrano B et al. ICO/IARC Information Centre on HPVand Cancer (HPV Information Centre). Human Papillomavirus and Related Diseases in the World. Summary Report 17 June 2019. [Accessed 15 October 2019].
2. Bray F, Ferlay J, Soerjomataram I et al. Global cancer statistics 2018: GLOBOCAN estimates of incidence and mortality worldwide for 36 cancers in 185 countries. A Cancer J Clin. 2018;68(6):394–424.
3. Ferlay J, Soerjomataram I, Ervik M et al. IARC Publications Website - GLOBOCAN 2012: Estimated Cancer Incidence, Mortality and Prevalence Worldwide in 2012 v1.0 [Internet]. WHO, International Research on Cancer. 2012 [cited 2019 Nov 18]. Available from: https://publications.iarc.fr/Databases/Iarc-Cancerbases/GLOBOCAN-2012-Estimated-Cancer-Incidence-Mortality-And-Prevalence-Worldwide-In-2012-V1.0-2012
4. Rudd P, Gorman D, Meja S et al. Cervical cancer in southern Malawi: A prospective analysis of presentation, management and outcomes. Malawi Med J. 2017;29(2):124–9.
5. Msyamboza KP, Ngwira B, D. The burden of selected chronic non-communicable diseases and their risk factors in malawi: Nationwide steps survey. PLoS One. 2011;6(5):6–11.
6. Itano J, Taoka KN. Core curriculum for oncology nursing. Elsevier Saunders; 2005. 970 p.
7. National Statistical O. 2018 Malawi Population and Housing Census Main Report [Internet]. 2019. Available from: http://10.150.72.21:6510/www.nsomalawi.mw/images/stories/data_on_line/demography/census_2018/2018 Malawi Population and Housing Census Main Report.pdf
8. Omoniyi-esan OG, Osasan SA, Ojo OS. Non-neoplastic diseases of the cervix in Nigerians : A histopathological study. African Heal Sci Vol. 2006;6(2):76–80.
9. Nwachokor FN, Forae GD. Morphological spectrum of non - neoplastic lesions of the uterine cervix in Warri , South - South , Nigeria. 2013;16(4):429–32.
10. Faduyile FA, Soyemi SS, Wright KO et al. Histopathological study of surgical cervical biopsies in Lagos ,. Trop J Obstet Gynaecol. 2017;34(2):124–8.
11. Dunne EF, Unger ER, Mcquillan G et al. Prevalence of HPV Infection Among Females in the United States. 2007;297(8):813–9.
12. Malawi Ministry of Health and Population, UNFPA. Malawi National cervical cancer control Strategy 2016-2020. Unfpa [Internet]. 2017;60. Available from: http://www.embase.com/search/results?subaction=viewrecord&from=export&id=L71343074%5Cnhttp://dx.doi.org/10.1158/1538-7445.AM2013-ADV01%5Cnhttp://fq5np7af6j.search.serialssolutions.com?sid=EMBASE&issn=00085472&id=doi:10.1158/1538-7445.AM2013-ADV01&atitle=N
13. Obaseki D, Nwafor C. Cervical Cancer Screening In Benin City, South-South Nigeria [Internet]. Vol. 5, IOSR Journal of Dental and Medical Sciences (IOSR-JDMS) e-ISSN. 2013 [cited 2019 Nov 18]. Available from: www.iosrjournals.org
14. Chukwuali LI, Onuigho WIB, Mgbor NC. Cervical cancer screening in Enugu, Nigeria. Trop J Obs Gynaecol. 2003;20:109–12.

For peer review only - http://bmjopen.bmj.com/site/about/guidelines.xhtml

15. Fonn S, Bloch B, Mabina M et al. Prevalence of pre-cancerous leisons and cervical cancer in South Africa-A Multicentre study. SAMJ. 2002;92(2):148–56.
16. Balaha MH, Moghannum MS Al, Ghowinem NA et al. Cytological pattern of cervical Papanicolaou smear in eastern region of Saudi Arabia. J Cytol. 2011;28(4):173–177.
17. Patel MM, Pandya AN, Modi J. Cervical pap smear study and its utility in cancer screening, to specify the strategy for cervical cancer control. Natl J COMMUNITY Med. 2011;2(1):49–51.
18. Insinga RP, Glass AG, Rush BB. Diagnoses and outcomes in cervical cancer screening: A population-based study. Am J Obstet Gynecol. 2004 Jul;191(1):105–13.
19. Okoye C. Histopathological pattern of cervical cancer in Benin City, Nigeria. J Med Investig Pract. 2014 Oct 1;9(4):147–147.
20. Pathak T, Pun C, Shrestha S et al. Incidence trends and histopathological pattern of cervical malignancies at BP koirala memorial centre hospital, Nepal. J Pathol Nepal. 2013;3:386–9.
21. Badar F, Anwar N, Meerza F et al. Cervical Carcinoma in a Muslim Community. Asian Pacific J Cancer Prev. 2007;8:24–6.
22. Olu-Eddo AN, Ekanem VJ, Umannah I et al. A 20 year histopathological study of cancer of the cervix in Nigerians. Nig Q J Hosp Med. 2011 Apr;21(2):149–53.
23. Chadza E, Chirwa E, Maluwa A et al. Factors that contribute to delay in seeking cervical cancer diagnosis and treatment among women in Malawi. 2012;4(11):1015–22.
24. Soares GR, Vieira R da R, Pellizzer EP et al. Indications for the HPV vaccine in adolescents: A review of the literature. J Infect Public Health [Internet]. 2015;8(2):105–16. Available from: http://dx.doi.org/10.1016/j.jiph.2014.08.011
25. Malawi Population-based HIV Impact Assessment Survey - MPHIA 2015 - 2016 [Internet]. Available from: https://phia.icap.columbia.edu/wp-content/uploads/2019/08/MPHIA-Final-Report_web.pdf
26. Abraham AG, Strickler HD, Jing Y et al. Invasive cervical cancer risk among HIV-infected women: A North American multi-cohort collaboration prospective study. J Acquir Immune Defic Syndr. 2013;62(4):405–13.
27. Ministry of Health and P. Malawi Guidelines for Clinical Management of HIV in Children and Adults (Third Edition, 2016) [Internet]. Lilongwe; 2016 [cited 2019 Nov 19]. Available from: www.hiv.health.gov.mw

For peer review only

Declarations

i. Ethical approval and consent to participant

For peer review only - http://bmjopen.bmj.com/site/about/guidelines.xhtml

This study was approved by National Health Science Research Committee as part of the main study "Pathological profile of malignancies in northern Malawi-A retrospective study at Mzuzu Central Hospital" number 19/05/2316. Both the Mzuzu Central Hospital Research and Publication Committee and the Mzuzu Central Hospital Laboratory department consents were obtained for the study. The need for informed consent was exempted from institutional review board due to the nature of the study, which involved a retrospective analysis of routinely collected data.

ii. **Consent for publication:** Not applicable

iii. **Availability of data and materials**

The datasets used and/or analyzed during the current study are available from the corresponding author on reasonable request. Email: kasekapaul2016@gmail.com

ORCID: 0000-0002-6651-8000

iv. **Competing interests:** The authors declare that they have no competing interest

v. **Funding**

The study's data collection, analysis, interpretation of data and manuscript writing was funded by Pingtung Christian Hospital, Taiwan through Luke International Norway (LIN), Malawi, Grant Number: PS-IR-108001

vi. **Authors contributions**

PUK and FWS conceived and designed the study. AK, CC, PK, JU and BCM contributed to development of the study protocol and supervised data collection and entry. AK analyzed the data and PUK drafted the manuscript. All authors read and approved the final manuscript.

vii. **Acknowledgements**

The authors are sincerely grateful to all the data collectors and the Laboratory manager for providing the data. The Pingtung Christian Hospital through Luke International Norway (LIN) is also appreciated for financial support

For peer review only - http://bmjopen.bmj.com/site/about/guidelines.xhtml

STROBE 2007 (v4) Statement—Checklist of items that should be included in reports of *cross-sectional studies*

Section/Topic	Item #	Recommendation	Reported on page #
**Title and abstract**	1	(*a*) Indicate the study's design with a commonly used term in the title or the abstract	1
		(*b*) Provide in the abstract an informative and balanced summary of what was done and what was found	1
**Introduction**			
Background/rationale	2	Explain the scientific background and rationale for the investigation being reported	3
Objectives	3	State specific objectives, including any prespecified hypotheses	3
**Methods**			
Study design	4	Present key elements of study design early in the paper	4
Setting	5	Describe the setting, locations, and relevant dates, including periods of recruitment, exposure, follow-up, and data collection	4
Participants	6	(*a*) Give the eligibility criteria, and the sources and methods of selection of participants	4
Variables	7	Clearly define all outcomes, exposures, predictors, potential confounders, and effect modifiers. Give diagnostic criteria, if applicable	4
Data sources/ measurement	8*	For each variable of interest, give sources of data and details of methods of assessment (measurement). Describe comparability of assessment methods if there is more than one group	4
Bias	9	Describe any efforts to address potential sources of bias	*4*
Study size	10	Explain how the study size was arrived at	4
Quantitative variables	11	Explain how quantitative variables were handled in the analyses. If applicable, describe which groupings were chosen and why	4
Statistical methods	12	(*a*) Describe all statistical methods, including those used to control for confounding	4
		(*b*) Describe any methods used to examine subgroups and interactions	5
		(*c*) Explain how missing data were addressed	5
		(*d*) If applicable, describe analytical methods taking account of sampling strategy	5
		(*e*) Describe any sensitivity analyses	5
**Results**			5

For peer review only

Participants	13*	(a) Report numbers of individuals at each stage of study—eg numbers potentially eligible, examined for eligibility, confirmed eligible, included in the study, completing follow-up, and analysed	5
		(b) Give reasons for non-participation at each stage	Not Applicable
		(c) Consider use of a flow diagram	Not Applicable
Descriptive data	14*	(a) Give characteristics of study participants (eg demographic, clinical, social) and information on exposures and potential confounders	5
		(b) Indicate number of participants with missing data for each variable of interest	5
Outcome data	15*	Report numbers of outcome events or summary measures	6
Main results	16	(*a*) Give unadjusted estimates and, if applicable, confounder-adjusted estimates and their precision (eg, 95% confidence interval). Make clear which confounders were adjusted for and why they were included	6
		(*b*) Report category boundaries when continuous variables were categorized	6
		(*c*) If relevant, consider translating estimates of relative risk into absolute risk for a meaningful time period	6
Other analyses	17	Report other analyses done—eg analyses of subgroups and interactions, and sensitivity analyses	8
**Discussion**			
Key results	18	Summarise key results with reference to study objectives	9
Limitations	19	Discuss limitations of the study, taking into account sources of potential bias or imprecision. Discuss both direction and magnitude of any potential bias	12
Interpretation	20	Give a cautious overall interpretation of results considering objectives, limitations, multiplicity of analyses, results from similar studies, and other relevant evidence	12
Generalisability	21	Discuss the generalisability (external validity) of the study results	12
**Other information**			
Funding	22	Give the source of funding and the role of the funders for the present study and, if applicable, for the original study on which the present article is based	15

*Give information separately for cases and controls in case-control studies and, if applicable, for exposed and unexposed groups in cohort and cross-sectional studies.

Note: An Explanation and Elaboration article discusses each checklist item and gives methodological background and published examples of transparent reporting. The STROBE checklist is best used in conjunction with this article (freely available on the Web sites of PLoS Medicine at http://www.plosmedicine.org/, Annals of Internal Medicine at http://www.annals.org/, and Epidemiology at http://www.epidem.com/). Information on the STROBE Initiative is available at www.strobe-statement.org.

For peer review only

BMJ Open

Histopathological Profile of Cervical Biopsies in Northern Malawi: A Retrospective Cross-sectional Study

Journal:	*BMJ Open*
Manuscript ID	bmjopen-2020-048283.R1
Article Type:	Original research
Date Submitted by the Author:	28-Sep-2021

Complete List of Authors:	Kaseka, Paul; Mzuzu Central Hospital,<br>Kayira, Alfred; Mzuzu Central Hospital<br>Chimbatata, Chikondi; Mzuzu Central Hospital<br>Chisale, Master; Mzuzu University, Biological Sciences; Mzuzu University<br>Kamudumuli, Pocha; University of Maryland and Global Initiative Company<br>Wu, Tsung-Shu; Ping Tung Christian Hospital<br>Mbakaya, Balwani; University of Livingstonia<br>Sinyiza, Frank; Mzuzu Central Hospital

**Primary Subject Heading**:	Oncology
Secondary Subject Heading:	Obstetrics and gynaecology, Pathology
Keywords:	ONCOLOGY, OBSTETRICS, Histopathology < PATHOLOGY, Adult oncology < ONCOLOGY, Gynaecological oncology < GYNAECOLOGY

SCHOLARONE™
Manuscripts

I, the Submitting Author has the right to grant and does grant on behalf of all authors of the Work (as defined in the below author licence), an exclusive licence and/or a non-exclusive licence for contributions from authors who are: i) UK Crown employees; ii) where BMJ has agreed a CC-BY licence shall apply, and/or iii) in accordance with the terms applicable for US Federal Government officers or employees acting as part of their official duties; on a worldwide, perpetual, irrevocable, royalty-free basis to BMJ Publishing Group Ltd ("BMJ") its licensees and where the relevant Journal is co-owned by BMJ to the co-owners of the Journal, to publish the Work in this journal and any other BMJ products and to exploit all rights, as set out in our licence.

The Submitting Author accepts and understands that any supply made under these terms is made by BMJ to the Submitting Author unless you are acting as an employee on behalf of your employer or a postgraduate student of an affiliated institution which is paying any applicable article publishing charge ("APC") for Open Access articles. Where the Submitting Author wishes to make the Work available on an Open Access basis (and intends to pay the relevant APC), the terms of reuse of such Open Access shall be governed by a Creative Commons licence – details of these licences and which Creative Commons licence will apply to this Work are set out in our licence referred to above.

Other than as permitted in any relevant BMJ Author's Self Archiving Policies, I confirm this Work has not been accepted for publication elsewhere, is not being considered for publication elsewhere and does not duplicate material already published. I confirm all authors consent to publication of this Work and authorise the granting of this licence.

For peer review only

Histopathological Profile of Cervical Biopsies in Northern Malawi: A Retrospective Cross-sectional Study

Paul Uchizi Kaseka[1*], Alfred Kayira[1], Chikondi Sharon Chimbatata[1], Master Chisale[1,2,3,] Pocha Kamudumuli[4], Tsung-Shu Joseph Wu[2,5], Balwani Chingatichifwe Mbakaya,[6,7]Frank Watson Sinyiza[1]

[1]Mzuzu Central Hospital, Mzuzu City, Malawi

[2]Luke International, Mzuzu City, Malawi

[3]Mzuzu University, Faculty of Sciences, Technology and Innovations, Biological Sciences, Mzuzu City, Malawi

[4]University of Maryland Global Initiative Corporation, Baltimore, Lilongwe City, Malawi

[5]Pingtung Christian Hospital, Taiwan

[6]University of Livingstonia, Department of Public Health, Malawi

[7]Mzuzu University, Faculty of Health Sciences, Mzuzu City, Malawi

*Corresponding author: kasekapaul2016@gmail.com

ABSTRACT

Objectives: According to the World Health Organization (2014), cervical cancer is the second most common cancer in women globally. More than 85% of the global cervical cancer morbidity and mortality occur in developing countries and the highest risk region is in Eastern and Southern Africa. Malawi has the highest age standardized rate of cervical cancer in the world. This study was carried out to determine the histopathological profile of cervical biopsies in a public tertiary hospital in Mzuzu, northern region of Malawi.

Setting: A public tertiary hospital in Mzuzu, northern region of Malawi

Participants: This was a retrospective study of all cervical biopsy specimen reports received in a public tertiary hospital in northern Malawi over a period of 5 years from July 2013-June 2018. Demographic, clinical and diagnostic data was obtained from original histopathology reports.

For peer review only - http://bmjopen.bmj.com/site/about/guidelines.xhtml

Results: A total of 500 cervical biopsy reports were reviewed during the study period. The mean age of the patients was 41.99±12.5. Age ranged from 15 to 80 years. Cervicitis accounted for 46.0% (n=162) of the total nonmalignant lesions seen, followed by cervical intraepithelial neoplasm (CIN), at 24.4% (n=86) and endocervical polyp, at 20.5% (n=72). Squamous cell carcinoma (SCC) accounted for 15.6% (n=78) of the total cervical biopsies studied and 85.7% of all total malignant lesions. Adenocarcinoma and undifferentiated carcinoma were 8.8% and 4.4% respectively of the total malignant diagnosis. All patients with malignant lesions had HIV.

Conclusion: Our study shows that cervicitis and squamous cell carcinoma were most common among nonmalignant and malignant cervical biopsies respectively. Since the frequency of cervical cancer is high, there is need to have well detailed national policies to be put in place to increase detection of pre-invasive lesions in order to reduce the prevalence of cervical cancer.

Key words: Cervical biopsies, cervical cancer, cervical intraepithelial neoplasm, cervicitis, malignant

Strengths and limitation of this study

- The study was able to determine the prevalence and associations of multiple exposures and outcomes.
- This being a retrospective cross-section study, the participants were neither deliberately exposed nor treated; thus, there were no ethical difficulties.
- This study depended on data that were entered into clinical database and not collected in a predesigned proforma as per specific requirements of the study as a result some records were excluded due to missing of certain crucial information.
- Since in retrospective studies, researchers have no control over the exposure of cases versus controls, these unrecognized confounders may have influenced the results.
- The study is a single-hospital-based review and as such inadequate to draw conclusions, but it does shed some light on pathological pattern of cervical cancer in Malawi.

For peer review only - http://bmjopen.bmj.com/site/about/guidelines.xhtml

BACKGROUND

Cervical cancer, after breast cancer, is the second most common cancer in women aged 15 to 44 years and it is the third leading cause of cancer in females worldwide(1). According to the International Agency for Research on Cancer(IARC) estimates, there are 570,000 new cases of cervical cancer annually, resulting into more than 311,000 deaths in 2018 globally(2). Most of the global burden lies in less developed countries, with sub-Saharan Africa (SSA) having the largest age-standardized incidence and mortality rates.Malawi has the highest cervical cancer incidence and mortality in the world with age-standardized rate (ASR) of 75.9 and 49.8 per 100,000 population respectively(3). World Health Organization (WHO) estimates suggest that every year there are at least 3,684 new cases of cervical cancer in Malawi and over 2,314 die from the disease(3).The exact number of cervical cancer morbidity and mortality among Malawian women, is not clear. This could partly be due to unrecorded or underreported cases because of the pathological based cancer registry not being maintained, and also lack of a national system of death certification(4). However, the 2010 National population-based cancer registry indicates that among females, cancer of the cervix was the commonest, accounting for 45.4% of all cases followed by Kaposi’s sarcoma (21.1%), cancer of the oesophagus (8.2%), breast cancer (4.6%) and non-Hodgkin lymphoma (4.1%)(5).

The Ministry of Health and Population of the government of Malawi, through the Sexual and Reproductive Health Directorate has implemented a cervical cancer screen-and-treat programme using visual inspection with acetic acid (VIA) approach since 2004, with women between 30-50 years as the main target (6). Women, no younger than 30 years, are offered three (3) free smears, with a 10 years interval in between each smear. Those screened for first time at the age of 55 or more have only one smear if the first smear is normal(6). Cervical screening using VIA is increasingly available in local clinics through the National Cervical Cancer Control Programme(6,7). From clinics, patients are referred to District Hospitals and/or Central Hospitals. At Mzuzu Central Hospital, women with suspected cervical cancer are managed at gynaecologyand/or oncology departments. Women with low-grade squamous intraepithelial lesions require

For peer review only - http://bmjopen.bmj.com/site/about/guidelines.xhtml

re-screening within a 12 months' period, whilst those with high-grade lesions are referred for colposcopy.

The standard method for diagnosis of cervical precancerous lesions is histopathological examination of tissue obtained through biopsy guided by colposcopy. When abnormalities are identified, cervical biopsy confirms the diagnosis of cancer(8).This study was carried out to determine the histopathological profile of cervical biopsies in a public tertiary hospital in Mzuzu, northern region of Malawi. The specific objectives were to determine the prevalence of both precancerous and cancerous cervical lesions; characterization of precancerous and cancerous lesions and risk factors of cervical cancer. Understanding the histological pattern of cervical cancer could guide development of focused preventive, care and treatment guidelines to address shortfalls in the care that is provided to cervical cancer patients in Malawi. Ultimately, this could contribute to the global target of 25% reduction of premature mortality from non communicable diseases (NCDs) by the year 2025(9).

MATERIALS AND METHODS

Design, setting and population

This was a retrospective study of all cervical biopsies reports received from Kamuzu Central Hospital/University of North Carolina (KCH/UNC) pathology laboratory in Lilongwe for Mzuzu Central Hospital (MCH), the only public tertiary hospital in the northern region of Malawi. This hospital does not have a functional pathology laboratory and relies on the KCH/UNC pathology laboratory for its services. The hospital is located in the northern part of Malawi catering for a population of about 2,289,780 million people(10)and serving 5 government District Hospitals, 3 Christian Health Association of Malawi (CHAM) hospitals and several private hospitals and clinics**.**

While some women presented to the gynaecological clinic asymptomatically through screening after VIA indicating a cancer, others presented with symptoms as referrals from a local clinic or other district hospitals from northern region. The most common symptoms were vaginal bleeding (often post coital), vaginal discharge with dysuria,

For peer review only - http://bmjopen.bmj.com/site/about/guidelines.xhtml

abdominal pain, vomiting and weight loss.

Tissue specimens were collected and preserved in 10% buffered formalin solution and then transported to Kamuzu Central Hospital/ University of North Carolina (KCH/UNC) pathology laboratory in Lilongwe. The KCH/UNC laboratory adheres to international quality assurance standards. The flow diagram (see Figure 1 attached) shows the process involved in coming up with a histopathological diagnosis at KCH/UNC laboratory in Lilongwe.

Within the 5-year period of the study (July 2013 to June 2018), a total of 2294 biopsy reports were received from KCH/UNC Histopathology laboratory.Data extracted included age, year, anatomic site, nature of specimen, clinical diagnosis, histopathological diagnosis, HIV status and whether the specimen was nonmalignant or malignant. Out of 2294 biopsy reports 500 were cervical biopsy reports representing 21.8% of the total biopsy reports received. In this study a total of 500 cervical cancer pathology reports were analyzed. Eleven reports which had missing demographic and clinical data or had inconclusive results were excluded.HIV status was defined as whether the patient was HIV sero reactive (positive), negative or not tested (unknown) when the biopsy was being taken. HPV status was defined as the patient sample being positive or negative upon histopathology examination.

Patient and public involvement

No patient involved

Data analysis

Data were entered in Microsoft excel 2016, validated and cleaned before importing into Stata, version 13.0 (Stata Corp. LP, College Station, TX, United States of America) for analysis. Descriptive analyses were performed to summarize patients' sociodemographic and clinical characteristics. A multivariate logistic regression was used to estimate the magnitude of the association between predictor variables (age and HIV status) and cancer at 95% confidence level.

For peer review only - http://bmjopen.bmj.com/site/about/guidelines.xhtml

Ethical clearance

This study was approved by National Health Science Research Committee (NHSRC) as part of the main study “Pathological profile of malignancies in northern Malawi-A retrospective study at Mzuzu Central Hospital”number 19/05/2316.Both the MCH Research and Publication Committee and the MCH Laboratory department consents were obtained for the study. The need for informed consent was exempted from the institutional review board due to the nature of the study, which involved a retrospective analysis of routinely collected data.

RESULTS

Within the 5-year period of the study (July 2013 to June 2018), a total of 500 biopsy reports were received from KCH/ UNC Pathology laboratory. The mean age of patients included in this study was 41.99 ± 12.5. The age range was 15-80 years. Most of the cervical biopsies were from patients in the 31-40 age group (34.8% n=174). Twelve percent of the cervical biopsies were from HIV positive patients and 51% of the results had unknown HIV result (Table 1).

Table 1. Demographic profile of cervical biopsies

**Parameter**		**Biopsies analysed**	
		**Frequency**	**Percentage (%)**
Age(Mean)years	20 and below (17)	3	0.6
	21-30(27)	57	11.4
	31-40(36)	174	34.8
	41-50(45)	132	26.4
	51-60(54)	84	16.8
	61-70(65)	29	5.8
	Above 70(75)	7	1.4
	Missing	14	2.8

For peer review only - http://bmjopen.bmj.com/site/about/guidelines.xhtml
For peer review only

HIV status	Positive	61	12.2
	Negative	184	36.8
	Unknown (not documented)	255	51.0

Of all the cervical biopsies studied, 91 (18.2%) were malignant. Squamous cell carcinoma (SCC) accounted for 85.7% of all malignant lesions (Table 2). Cervicitis accounted for 46.0% (n=162) of the total nonmalignant lesions seen. Ten percent of cervicitis cases and 30.2% of CIN cases had HPV respectively (Table 2).

Table 2. Histopathological diagnosis of cervical biopsies

Parameter	Frequency	Within group percentage(%)	Percentage(%)total
**Normal cervical tissue**	**46**		**9.2**
**Nonmalignant lesions**	**352**		**70.4**
Cervicitis*	162	46.0	32.4
Cervical intraepithelial neoplasm (CIN)*	86	24.4	17.2
Endocervical polyp	72	20.5	14.4
Carcinoma in situ	4	1.1	0.8
Nabothian cyst	4	1.1	0.8
Schistosomiasis	5	1.4	1.0
Condyloma (warts)	11	3.1	2.2
Others	8	2.3	1.6
**Malignant**	**91**		**18.2**
Squamous cell carcinoma	78	85.7	15.6
Adenocarcinoma	8	8.8	1.6
Undifferentiated	4	4.4	0.8
Carcinosaroma	1	1.1	0.2
**Inconclusive result**	**11**		**2.2**

For peer review only - http://bmjopen.bmj.com/site/about/guidelines.xhtml

Total	500	100

*Cervicitis with HPV= 10% (16/162)*CIN with HPV= 30.2% (26/86)

*CIN 1 = 34.9% (30/86), CIN 2 = 22.1% (19/86), CIN 3 = 43.0% (37/86)

The chances of developing cervical cancer increased with advancing age, with the risk becoming more prominent after age 60 years. Women aged 61-70 years were 6.2 times (OR = 6.2, 95% CI: 2.1 - 18.4) more likely to develop cervical cancer than those in the 21-30 years' age category. The odds of getting cancer were higher in women living with HIV. Women with HIV were more than twice (OR = 2.3, 95% CI: 1.1 - 4.9)as likely to have cervical cancer as those who were HIV negative (Table 3).

For peer review only

For peer review only - http://bmjopen.bmj.com/site/about/guidelines.xhtml

Table 3: Association between cancer, age and HIV status

		Cancer (%)	Non cancer (%)	Unadjusted odds ratio (95% CI)†	Adjusted odds ratio (95% CI)†
Age (years)	20 and below	0 (0.0)	2 (100.0)		
	21 - 30 (reference)	7 (12.3)	50 (87.2)	-	
	31 - 40	25 (14.6)	146 (85.4)	1.2 (0.5 -3.0)	1.2 (0.5- 2.9)
	41 - 50	23 (17.7)	107 (82.3)	1.5 (0.6 - 3.8)	1.5 (0.6 - 3.8)
	51 - 60	18 (22.2)	63 (77.8)	2.0 (0.8 - 5.3)	2.2 (0.8 - 5.7)
	61 - 70	13 (44.8)	16 (55.2)	5.8 (2.0 - 17.0)	6.2 (2.1 - 18.4)
	Above 70	1 (20.0)	4 (80.0)	1.8 (0.2 - 18.3)	1.9 (0.2 - 19.9)
HIV status	Negative (reference)	26 (14.4)	155 (85.6)	-	
	Positive	15 (24.6)	46 (75.4)	1.9 (1.0 - 4.0)	2.3 (1.1 - 4.9)
	Unknown	50 (20.2)	197 (79.8)		

Fourteen patients (4 cancer cases and 10 non cancer cases) had no data on age and were excluded from this analysis

†Binary logistic regression

For peer review only - http://bmjopen.bmj.com/site/about/guidelines.xhtml

DISCUSSION

To the best of our knowledge, this is the first study conducted to determine the histopathological profile of cervical biopsies in a public tertiary hospital in Mzuzu, northern region of Malawi. This study found more benign conditions (70.4% n=352) than neoplastic malignant conditions (18.2% n=91). This is in contrast with other studies undertaken in developing countries which reported more neoplastic malignant conditions than benign conditions(8,9).

Our study found that cervicitis, an inflammatory disease comprising acute cervicitis and chronic non-specific cervicitis was the most common nonmalignant condition.It accounted for 46% of all the nonmalignant tumours and 32.4% of all cervical biopsies received in this study. This was consistent to a study conducted in Nigeria which had 37.5% cervicitis of all cervical biopsies included in that study(11). Most cases of cervicitis are often due to non-specific causes or infective agents(12). Cervicitis has been highly reported in previously studies in other countries(12,13).

In this study CIN, a premalignant lesion ranked second in nonmalignant lesions accounting for 24% of all nonmalignant lesions and 17.2% of all cervical biopsies with the prevalence of CIN I, CIN II, CIN III of 34.9%, 22.1% and 43% respectively. This finding is comparable to a study conducted in Nigeria which reported CIN constituting 15% of all cervical biopsies reviewed(13). In that study however, there was a reduction in the prevalence of CIN from low grade CIN to high grade CIN(13). In our study, the high grade CIN (CIN III) was the most common. This finding indicates that invasive cervical cancer progresses from advanced stages of precancerous lesions. This shows that there is need to create community awareness and strengthen early cervical cancer screening for Malawi to have better outcomes.

In the current study 10% (n=16) of all the cervicitis cases and 30.2% (n=26) of all the CIN biopsies had HPV. This finding suggests that there is high rate of HPV infections, the causative agent of CIN in the younger age group(13). Unfortunately, the data on population-based, age specific prevalence of HPV are not available in Malawi. However, WHO estimates that the overall HPV prevalence in Malawi is about 34%(7).HPV

For peer review only - http://bmjopen.bmj.com/site/about/guidelines.xhtml

infection and precancerous lesions are usually difficult to notice and develop into full blown cancer before women realize the need to seek medical care.

The rate of malignant lesions (tumours) in our study (18.2%) is comparable to 16.2% and 12.2% in studies conducted in Benin City(14), and in Enugu, in Nigeria(15) respectively. Studies done in South Africa, Saudi Arabia, India and United States, with malignant lesions at 2.42%, 4.95%, 5.5% and 5.0% respectively, are all at variance with the current study which explains that early cervical cancer screening helps to reduce the prevalence of cervical cancer(16–19).

Among the malignant tumours, SCC was the most common histological type of cervical cancer in our study. SCC accounted for 85.7% of malignant lesions cervical cancer and was also the most common diagnosis, at 15.6% of all cervical biopsies in this study. This is consistent with findings from other studies conductedworldwide (20–22). As Faduyile et al. observed the high rate of SCC in Malawi and Africa could reflect the low uptake of VIA and Pap smear test which are capable of identifying dysplastic conditions before transformation to malignancy(11). This shows that there is need for Malawi to have well organized cervical cancer screening and Pap smear test to reduce the prevalence of SCC(2).

In our study, adenocarcinorma and poorly differentiated carcinorma were 8.8% and 4.4% respectively of the total malignant diagnosis. The prevalence of adenocarcinoma and poorly differentiated carcinoma observed in this study is in contrast to other studies done in Nigeria where one study found 5.8% and 2.0% of the total malignant lesions diagnosis and another found 6.0% and 1.0% respectively(13,23). The high prevalence found in this study confirms a previous study findings by Chanza et al.(24). Chanza et al. reported that Malawian women delay to seek medical attention due to limited knowledge on symptoms and signs, limited financial resources, limited accessibility and unavailability of cancer screening facilities hence late diagnosis. These results show that early cervical cancer screening, increased awareness, better health care facilities, accessibility, improved histopathological confirmatory diagnosis and early treatment by surgeons, may reduce the cervical cancer burden in Malawi.

For peer review only - http://bmjopen.bmj.com/site/about/guidelines.xhtml

In the current study it has been observed that the diagnosis of cervical cancer was significantly associated with the age of the patients. The chances of developing cervical cancer increased with advancing age, with the risk becoming more prominent after age 60 years. Women aged 61-70 years were 6.2 times (OR = 6.2, 95% CI: 2.1 - 18.4)more likely to develop cervical cancer than those in the 21-30 years age category(Table 3). It is important to note that high quality screening programs are important to prevent cervical cancer among unvaccinated older women(25). None of the women in our study had received any HPV vaccine in their lifetime as the first round of the mass HPV vaccine in this country was administered in January 2019 mainly in schools, targeting 9-13 year old girls who had not yet become sexually active and the second round was given in January 2020. In Malawi, the integration of HPV vaccine programs with adequate screening programs in older women (aged 30-49 years) has the potential to reduce the burden of cervical cancer.

Malawi's HIV prevalence is one of the highest in the world, with 10.6% of adult population (aged 15-64) living with HIV(26). With such a high HIV prevalence in Malawi, there is an increased risk of AIDS-defining cancers including cervical cancer. In this study the probability of getting cancer were higher in women living with HIV. Women with HIV were more than twice (OR = 2.3, 95% CI: 1.1 - 4.9)as likely to have cervical cancer as those who were HIV negative (Table 3).This could be attributed to the reason that HIV-infected women are more likely than HIV-uninfected women to have incident and persistent HPV cervical infections(27).A twelve monthly cervical cancer screening, increased availability of dolutegravir (DTG) based antiretroviral treatment (ART) to HIV infected women now being provided in the country, routine viral load checks (at 6 months, 12 months and every 12 months since initiation) and timely switch to second line or third line regimens to ensure viral load suppression will eventually have an impact on benign lesions progressing to invasive cervical cancer(28). However, in this study almost half (52.74%, n=48) of all the women diagnosed with cervical cancer had an unknown HIV status. This calls for scaling up of HIV testing in cancer screening settings for early diagnosis and ART referral and further research is warranted on barriers among HIV-infected women to seeking cancer screening services despite already being in the health care system.

For peer review only - http://bmjopen.bmj.com/site/about/guidelines.xhtml

LIMITATIONS

This study used available programme health facility data and histopathological reports on cervical cancer. The use of health facility data has its own limitations, such as incompleteness and bias in the sense that information is obtained only from people who came to the facility and underwent biopsy, leaving out those that did not seek medical care and or were not biopsied and therefore cannot be generalized to the general population.

The other limitation of this study is that it is a single-hospital-based review and as such inadequate to draw conclusions, but it does shed some light on pathological pattern of cervical cancer in Malawi.

Finally, this is a retrospective study so we could not be able to extract details for example in cases where tumours were diagnosed by screening or symptoms, presence or absence of the patient co-morbidities. Nevertheless, the comprehensive histopathological pattern of cervical cancer demonstrated by this study provides evidence that could be used to inform policies, strategies and intervention for prevention of cancer in Malawi.

CONCLUSION

The SCC was the commonest malignant condition and cervicitis and CIN were the most common non malignant conditions in all the women studied. Since the frequency of cervical cancer is high, there is need for well detailed national policies to be put in place to increase detection of pre-invasive lesions, which in turn will decrease the frequency of cervical cancer in the country. The presence of chronic non-specific cervicitis in women of reproductive age is infective in origin with its attending sequelae. Intensifying screening programmes among women and provision of long term ART to the HIV infected may offer an opportunity for appropriate interventions to reduce morbidity, mortality and reduce complications among these women.

Declarations

i. Ethical approval and consent to participant

For peer review only

This study was approved by National Health Science Research Committee as part of the main study "Pathological profile of malignancies in northern Malawi-A retrospective study at Mzuzu Central Hospital" number 19/05/2316.Both the Mzuzu Central Hospital Research and Publication Committee and the Mzuzu Central Hospital Laboratory department consents were obtained for the study. The need for informed consent was exempted from institutional review board due to the nature of the study, which involved a retrospective analysis of routinely collected data.

ii. **Consent for publication:** Not applicable

iii. **Availability of data and materials**

The datasets used and/or analyzed during the current study are available from the corresponding author on reasonable request. Email: kasekapaul2016@gmail.com

ORCID: 0000-0002-6651-8000

iv. **Competing interests:** The authors declare that they have no competing interest

v. **Funding**

The study's data collection, analysis, interpretation of data and manuscript writing was funded by Pingtung Christian Hospital, Taiwan through Luke International Norway (LIN), Malawi, Grant Number: PS-IR-108001

vi. **Authors contributions**

PUK and FWS conceived and designed the study. AK, CC, PK, TJW, MC and BCM contributed to development of the study protocol and supervised data collection and entry. AK analyzed the data and PUK drafted the manuscript. All authors read and approved the final manuscript.

vii. **Acknowledgements**

The authors are sincerely grateful to all the data collectors and the Laboratory manager for providing the data. The Pingtung Christian Hospital through Luke International Norway (LIN) is also appreciated for financial support

For peer review only - http://bmjopen.bmj.com/site/about/guidelines.xhtml

REFERENCES

1. Bruni L, Albero G, Serrano B et al. ICO/IARC Information Centre on HPVand Cancer (HPV Information Centre). Human Papillomavirus and Related Diseases in the World. Summary Report 17 June 2019.www.hpvcentre.net [Accessed 15 October 2019].
2. Bray F, Ferlay J, Soerjomataram I et al. Global cancer statistics 2018: GLOBOCAN estimates of incidence and mortality worldwide for 36 cancers in 185 countries. A Cancer J Clin. 2018;68(6):394–424.DOI: 10.3322/caac.21492.
3. Ferlay J, Soerjomataram I, Ervik M et al. IARC Publications Website - GLOBOCAN 2012: Estimated Cancer Incidence, Mortality and Prevalence Worldwide in 2012 v1.0 [Internet]. WHO, International Research on Cancer. 2012ISBN-13 (Database) 978-92-832-2447-1 [cited 2019 Nov 18]. Available from: https://publications.iarc.fr/Databases/Iarc-Cancerbases/GLOBOCAN-2012-Estimated-Cancer-Incidence-Mortality-And-Prevalence-Worldwide-In-2012-V1.0-2012
4. Rudd P, Gorman D, Meja S et al. Cervical cancer in southern Malawi: A prospective analysis of presentation, management and outcomes. Malawi Med J. 2017;29(2):124–9.PMID: 28955419.
5. Msyamboza KP, Ngwira B, D. The burden of selected chronic non-communicable diseases and their risk factors in malawi: Nationwide steps survey. PLoS One. 2011;6(5):6–11.DOI.org/10.1371/journal.pone.0020316https://doi.org/10.1371/journal.pone.0020316.
6. Msyamboza KP, Phiri T, Sichali W, Kwenda W, Kachale F. Cervical cancer screening uptake and challenges in Malawi from 2011 to 2015: Retrospective cohort study. BMC Public Health [Internet]. 2016;16(1):1–6. Available from: http://dx.doi.org/10.1186/s12889-016-3530-y
7. Malawi Ministry of Health and Population, UNFPA. Malawi National cervical cancer control Strategy 2016-2020 [Internet]. Unfpa. 2017. Available from: http://www.embase.com/search/results?subaction=viewrecord&from=export&id=L71343074%5Cnhttp://dx.doi.org/10.1158/1538-7445.AM2013-ADV01%5Cnhttp://fq5np7af6j.search.serialssolutions.com?sid=EMBASE&issn=00085472&id=doi:10.1158/1538-7445.AM2013-ADV01&atitle=N
8. Itano J, Taoka KN. Core curriculum for oncology nursing. Elsevier Saunders; 2005. 970 p.
9. Noncommunicable diseases country profiles 2018. Geneva: World Health Organization; 2018. Licence: CC BY-NC-SA 3.0 IGO.
10. National Statistical O. 2018 Malawi Population and Housing Census Main Report [Internet]. 2019. Available from: http://10.150.72.21:6510/www.nsomalawi.mw/images/stories/data_on_line/demography/census_2018/2018 Malawi Population and Housing Census Main Report.pdf
11. Omoniyi-esan OG, Osasan SA, Ojo OS. Non-neoplastic diseases of the cervix in Nigerians : A histopathological study. African Heal Sci Vol. 2006;6(2):76–80.PMCID: PMC1831974 PMID: 16916295.

For peer review only - http://bmjopen.bmj.com/site/about/guidelines.xhtml

12. Nwachokor FN, Forae GD. Morphological spectrum of non - neoplastic lesions of the uterine cervix in Warri , South - South , Nigeria. 2013;16(4):429–32.DOI: 10.4103/1119-3077.116883.
13. Faduyile FA, Soyemi SS, Wright KO et al. Histopathological study of surgical cervical biopsies in Lagos ,. Trop J Obstet Gynaecol. 2017;34(2):124–8.DOI: 10.4103/TJOG.TJOG_28_17.
14. Obaseki D, Nwafor C. Cervical Cancer Screening In Benin City, South-South Nigeria [Internet]. Vol. 5, IOSR Journal of Dental and Medical Sciences (IOSR-JDMS) e-ISSN. 2013 [cited 2019 Nov 18]. e-ISSN: 2279-0853, p-ISSN: 2279-0861. www.iosrjournals.org. Available from: www.iosrjournals.org
15. Chukwuali LI, Onuigho WIB, Mgbor NC. Cervical cancer screening in Enugu, Nigeria. Trop J Obs Gynaecol. 2003;20:109–12.DOI: 10.4314/tjog.v20i2.14412.
16. Fonn S, Bloch B, Mabina M et al. Prevalence of pre-cancerous leisons and cervical cancer in South Africa-A Multicentre study. SAMJ. 2002;92(2):148–56. PMID: 11894653.
17. Balaha MH, Moghannum MS Al, Ghowinem NA et al. Cytological pattern of cervical Papanicolaou smear in eastern region of Saudi Arabia. J Cytol. 2011;28(4):173–177. DOI: 10.4103/0970-9371.86343

. 18. Patel MM, Pandya AN, Modi J. Cervical pap smear study and its utility in cancer screening, to specify the strategy for cervical cancer control. Natl J COMMUNITY Med. 2011;2(1):49–51. ISSN: 0976 3325.
19. Insinga RP, Glass AG, Rush BB. Diagnoses and outcomes in cervical cancer screening: A population-based study. Am J Obstet Gynecol. 2004 Jul;191(1):105–13. https://doi.org/10.1016/j.ajog.2004.01.043.
20. Okoye C. Histopathological pattern of cervical cancer in Benin City, Nigeria. J Med Investig Pract. 2014 Oct 1;9(4):147–147. link.gale.com/apps/doc/A414542015/AONE?u=nysl_ca_ind&sid=googleScholar&xid=7c182fa6. Accessed 18 Aug. 2021. Gale Document Number: GALE|A414542015.

21. Pathak T, Pun C, Shrestha S et al. Incidence trends and histopathological pattern of cervical malignancies at BP koirala memorial centre hospital, Nepal. J Pathol Nepal. 2013;3:386–9. DOI: https://doi.org/10.3126/jpn.v3i5.7863.
22. Badar F, Anwar N, Meerza F et al. Cervical Carcinoma in a Muslim Community. Asian Pacific J Cancer Prev. 2007;8:24–6. PMID: 17477766.
23. Olu-Eddo AN, Ekanem VJ, Umannah I et al. A 20 year histopathological study of cancer of the cervix in Nigerians. Nig Q J Hosp Med. 2011 Apr;21(2):149–53. PMID: 21913514.
24. Chadza E, Chirwa E, Maluwa A et al. Factors that contribute to delay in seeking cervical cancer diagnosis and treatment among women in Malawi. 2012;4(11):1015–22. DOI: 10.4236/health.2012.411155.
25. Soares GR, Vieira R da R, Pellizzer EP et al. Indications for the HPV vaccine in adolescents: A review of the literature. J Infect Public Health [Internet]. . 2015;8(2):105–16. Available from: http://dx.doi.org/10.1016/j.jiph.2014.08.011. http://dx.doi.org/10.1016/j.jiph.2014.08.011.

For peer review only - http://bmjopen.bmj.com/site/about/guidelines.xhtml

26. Malawi Population-based HIV Impact Assessment Survey - MPHIA 2015 - 2016 [Internet]. Available from: https://phia.icap.columbia.edu/wp-content/uploads/2019/08/MPHIA-Final-Report_web.pdf
27. Abraham AG, Strickler HD, Jing Y et al. Invasive cervical cancer risk among HIV-infected women: A North American multi-cohort collaboration prospective study. J Acquir Immune Defic Syndr. 2013;62(4):405–13. doi: 10.1097/QAI.0b013e31828177d7.
28. Ministry of Health and P. Malawi Guidelines for Clinical Management of HIV in Children and Adults (Third Edition, 2016) [Internet]. Lilongwe; 2016 [cited 2019 Nov 19]. Available from: www.hiv.health.gov.mw

For peer review only

Figure 1. Process involved in specimen processing at KCH/UNC laboratory

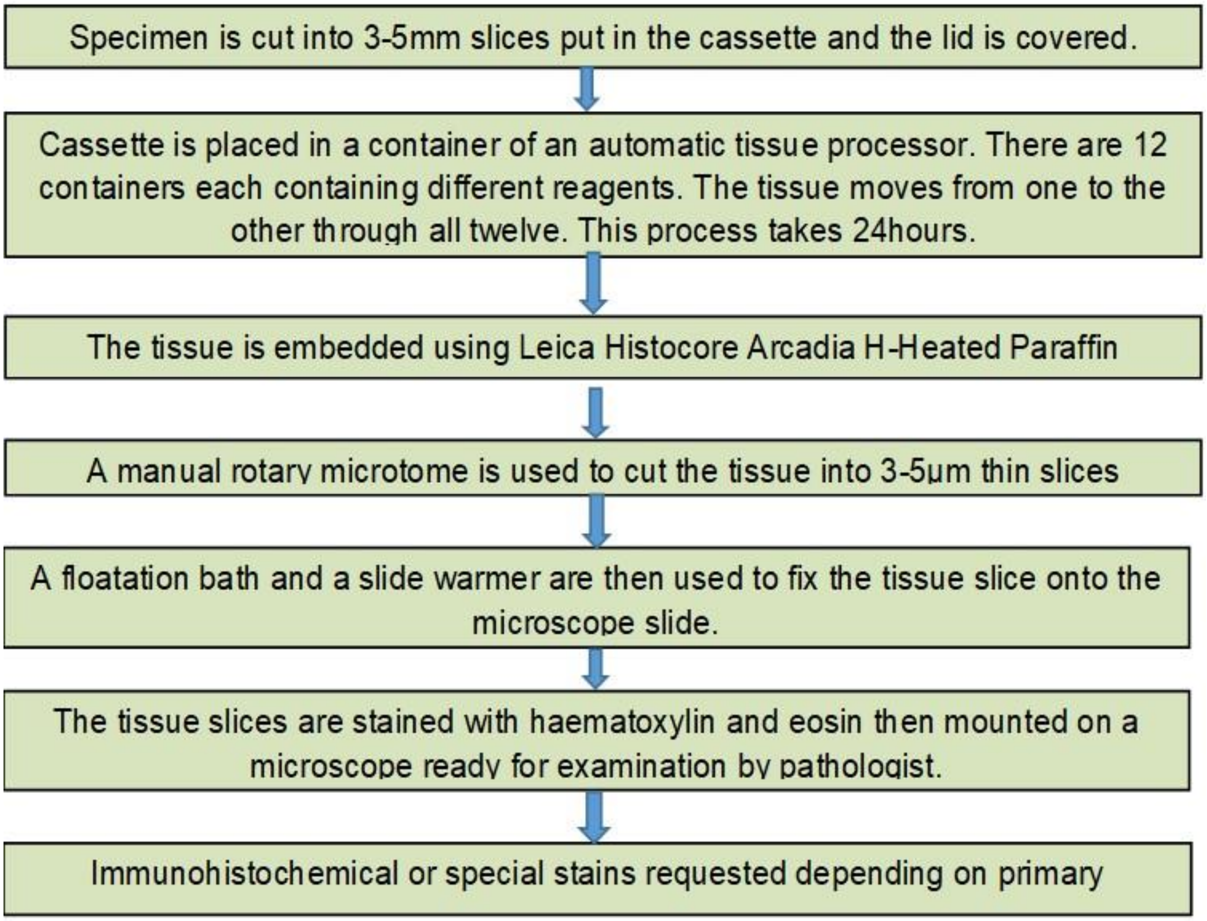

For peer review only - http://bmjopen.bmj.com/site/about/guidelines.xhtml

Authors responses to reviewers comments

Title: Histopathological Profile of Cervical Biopsies in Northern Malawi: A Retrospective Study

Authors:

Paul UchiziKaseka

Alfred Kayira

Chikondi Sharon Chimbatata

Master R.O Chisale

Pocha Kamudumuli

Tsung-Shu Joseph Wu

Balwani Chingatichifwe Mbakaya

Frank Watson Sinyiza

Version: 1

Date: 10th September, 2021

Dear Dr. Shona Reeves and all reviewers at BMJ Open

bmjopen-2020-048283: Histopathological Profile of Cervical Biopsies in Northern Malawi: A Retrospective Cross-Sectional Study

Editor/reviewers	Editor/Reviewers Comments	Authors Response
Comments from the Editor	- Please include the study design in the Title and the Abstract	Thank you so much for your comment. Study design has been included in title and abstract. See details on page 1
	Please revise the Strengths and Limitations section of your manuscript (after the Abstract). This section should contain five short bullet points, no longer than one sentence each, that relate specifically to the methods. Please do not	Thank you for your comment. This has been revised. See details on page 2

For peer review only

	discuss the results or impact of the study in this section.	
	- Formatting Amendments (where applicable):	
Reviewer: 1 Dr. Luz Alcántara-Quintana, UASLP	It is necessary to do this type of retrospective work, to publicize the cervical characteristics of the patients. As well as unifying criteria between pathologists.	Thank you so much for your comment.
Reviewer: 2 Dr. Maaike Bleeker, VU University Medical Centre Amsterdam	The paper Histopathological profile of cervical biopsies in Northern Malawi: A Retrospective Study aims determination of the histopathological outcome of 500 cervical biopsies over a period of 5 years in Northern Malawi. Information on the distribution on cervical diseases in this country is very important as the incidence of cervical cancer is one of the highest in the world. The data which are presented are moderate to clearly presented though there are multiple misspellings throughout the paper and there are several issues that deserves more attention or should be improved. Specific points:	Thank you so much for your comment
	**Abstract**<br>1) Give more clearly (1-2 sentences) the rationale behind the research aim of this study. To save words in the abstract, the 11 excluded reports can be left out her (and moved to the result section). This is well presented in the 'strengths'	Thank you so much for your comment. See details on page 1
	2) Include not only the SCC but also the other	Thank you so much for your

For peer review only

	malignant tumors (adenocarcinoma etc)	comment. A sentence on adenocarcinoma and undifferentiated carcinoma has been added. See details on page 2.
	3) 'All malignant tumours had HIV' is a strange sentence as HIV is not mentioned before and tumours do not have HIV. Patients can have HIV. If you want to give this information than you should also give an impression for the HIV status in the population studied and in de different disease groups.	Thank you so much for your comment. This sentence has been corrected to reflect patients and not biopsies. See details on page 2.
	4) Limitation: I think that the first point mentioned is not a limitation as this is a reflection of the current situation in Malawi. Although this situation limits the success of the cervical cancer reduction, this is not primarily a limitation of the study.	Thank you so much for your comment<br><br>All limitations have been revised as per comment from the editor. See details on page 2
	**Introduction**<br>5) The authors should give some more information on how current screening in Malawi is organized. Is VIA used (see-and-treat management)? Are cervical scrapes performed?	Thank you so much for your comment.<br><br>Information on how current screening in Malawi is organized has been added. See details on page 3 and 4.
	**Methods**<br>6) It should be clearly stated if the 500 biopsies analyzed were of 500 different patients or not. Do these also include multiple reports (biopsy and resection or LLETZ) from one	Thank you so much for your comment<br><br>In this study a total of 500 individual patient cervical cancer

For peer review only

	patient?	pathology reports were analysed. See details on page 4 to 5.
	7) Could you elaborate a bit more on how the cervical samples were retrieved? Through which pathways do patients arrive in the clinic? Are these women all walk-ins with gynecological complaints? Or is there any way of screening with pap-smears after which women are referred for colposcopy with cervical biopsy?	Thank you so much for your comment<br><br>Pathway has been described. See details on page 4 to 5.
	8) It was mentioned that beside age and year, also the nature of the specimen and the clinical diagnosis was recorded? Could this be stated more clearly? Nature (it were all cervical biopsies or do they mean for instance a clockwise indication, for instance biopsy at 12 o'clock?); Clinical diagnosis (what is meant? Can they present these results),	Thank you so much for your comment. This has now been clarified in the methods. See details on page 5.
	9) How was the HIV status and the HPV status defined?	HIV status was defined as whether the patient was HIV sero reactive (positive), negative or not tested (unknown) when the biopsy was being taken.<br><br>HPV status was defined as the patient sample was being positive or negative upon histopathology examination. These details have been provided in the methods. See details on page 5.
	**Results**<br>10) T1 can be completed with all clinical and	Thank you so much for the comment. More information has

For peer review only

	demographical data (as summarized in the methods). Include also mean age with subheadings of the age-groups	been added to methods section to clarify what happened. Mean age for all age groups has however been added in T1. See details on page 6
	11) T2 shows the histopathological diagnosis of cervical biopsies. The outline of the words Malignant and inconclusive seems off, should maybe be the same as normal cervical tissue and nonmalignant tissue. Adenocarcinoma (misspelled adenocarcioRma) should probably be outlined the same as squamous cell carcinoma.	Thank you very much for your comment.<br>The outline of the words have been corrected.<br>Adenocarcinoma spelling has been corrected. See details on page 7.
	12) In the statistical analysis you report using Chi square and Fisher's exact test to look for significant associations, but you do not report any comparisons / p-values? This should be added.	Thank you so much for your observation. The Ch2 of Fisher's exact test were left there in error. They were initially included in the methods section of the proposal. At that time the thinking was that we would find many variables from the records that we reviewed. In that case we planned to use a Chi2 or Fisher's exact test as appropriate to explore and isolate variables that were significantly associated with cancer at alpha (significant level) of 0.05 or less. Only those variables demonstrating a significant association with cancer at 95% confidence level would be carried forward for analysis in a

		multivariable logistic regression so as to quantify the association. Unfortunately, data was available only on two predictor variables – age and HIV status. Because these variables were toofew we just included both of them in the logistic regression at once. We have thus removed Chi2 or Fisher' exact test from the test. Please see page number 5.
	13) What is the total number of patients included for the analysis in T3? In T 2 you report 91 malignant cases, but the sum of 7+25+23+18+13+1 = 87 (for the age analysis). Same for the non-cancer cases, which is 46+352 = 398 in T2. In the age analysis of T3 the sum of non-cancer cases is n = 388. Was age data not available for all cases or why are there cases missing? Please clarify this point.	Thank you for your observation. The missing cases have now been accounted for. See footnote of Table 3 on page 9.
	14) T2 mentions only 30.2% HPV positivity in CIN lesions. This seems quite low. How is these HPV positivity rate in the different CIN groups? Do the high-grade CIN lesions have a higher HPV positivity rate? Or could there be another reason why the HPV positivity rate is so low? See also comment 9: I think when HPV is scored as 'viral changes' at histopathology that this information is not informative when you want to compare noncancer with cancer cases.	HPV positivity rate in the different CIN groups (i.e. CIN I, CIN II, CIN III) has been provided as a footnote of T2 on page 8.<br><br>CIN III had higher HPV positivity of 43.0%

For peer review only - http://bmjopen.bmj.com/site/about/guidelines.xhtml

	15) T2: check for misspellings (Nabothian cyst, Condyloma, carcinoma, carcinosaroma).	Thank you very much for the comment. Spellings for Nabothian cyst, Condyloma, carcinoma, carcinosaroma have corrected. Details are on page 8.
	16) T2: Poorly differentiated is not a category (it goes always with either SCC or adenocarcinoma), if not clear consider either the term undifferentiated or carcinoma NOS	Thank you very much for the comment. The term "poorly differentiated" has been replaced with "undifferentiated". Refer to page 8
	17) Are gynaecologists and/or pathologists involved in this paper?	Thank you very much for the comment. No gynaecologists and/or pathologists is involved in this paper
	18) Present consistently with one decimal (instead of alternative 1 or 2)	Thank you very much for the comment. One decimal has been used throughout the paper
	**Discussion**<br>19) In the discussion section you refer to reference number 11, a study in which the HPV prevalence in the united states among women aged 14 to 59 was evaluated. However, a big difference with your data is that this prevalence is evaluated within the general population, and not within a population of women with CIN. Interpretations should be more careful, see also comment 9 and 14.	Thank you very much for the comment.<br>Reference has been removed. See details on pages 10 to 12.
	20) Page 10, line 32: nonmalignant tumour. The term tumour is not justified for CIN I, II or III	Term "tumour" has been replaced with "lesion". See details on pages

For peer review only

		10
	21) Page 11, line 26: 85% SCC and 15% adenocarcinoma is more or less worldwide (and not only in Malawi, Nepal and Pakistan). It is not clear why a comparison with these 2 countries is made.	Reference has been removed. See details on page 11.
Reviewer: 3 Dr. Mona Lisa, All India Institute of Medical Sciences - Deoghar	There are some spelling and grammatical errors which I have marked in the file attached. I have also mentioned in the sticky notes some of my queries. Please answer them in the manuscript and resubmit. DOI should be added to the references.	Thank you very much for your comments.<br>Spellings have been corrected.<br>Issues raised in the manuscripts have been responded to accordingly.<br><br>DOI has been added to references. See details on page 15-17.

For peer review only

STROBE 2007 (v4) Statement—Checklist of items that should be included in reports of *cross-sectional studies*

**Section/Topic**	**Item #**	**Recommendation**	**Reported on page #**
**Title and abstract**	1	(*a*) Indicate the study's design with a commonly used term in the title or the abstract	1
		(*b*) Provide in the abstract an informative and balanced summary of what was done and what was found	1
**Introduction**			
Background/rationale	2	Explain the scientific background and rationale for the investigation being reported	3
Objectives	3	State specific objectives, including any prespecified hypotheses	4
**Methods**			
Study design	4	Present key elements of study design early in the paper	4
Setting	5	Describe the setting, locations, and relevant dates, including periods of recruitment, exposure, follow-up, and data collection	4
Participants	6	(*a*) Give the eligibility criteria, and the sources and methods of selection of participants	5
Variables	7	Clearly define all outcomes, exposures, predictors, potential confounders, and effect modifiers. Give diagnostic criteria, if applicable	5
Data sources/ measurement	8*	For each variable of interest, give sources of data and details of methods of assessment (measurement). Describe comparability of assessment methods if there is more than one group	5
Bias	9	Describe any efforts to address potential sources of bias	*5*
Study size	10	Explain how the study size was arrived at	5
Quantitative variables	11	Explain how quantitative variables were handled in the analyses. If applicable, describe which groupings were chosen and why	5
Statistical methods	12	(*a*) Describe all statistical methods, including those used to control for confounding	5
		(*b*) Describe any methods used to examine subgroups and interactions	5
		(*c*) Explain how missing data were addressed	5
		(*d*) If applicable, describe analytical methods taking account of sampling strategy	5
		(*e*) Describe any sensitivity analyses	5
**Results**			6

For peer review only

Participants	13*	(a) Report numbers of individuals at each stage of study—eg numbers potentially eligible, examined for eligibility, confirmed eligible, included in the study, completing follow-up, and analysed	6
		(b) Give reasons for non-participation at each stage	Not Applicable
		(c) Consider use of a flow diagram	Not Applicable
Descriptive data	14*	(a) Give characteristics of study participants (eg demographic, clinical, social) and information on exposures and potential confounders	6
		(b) Indicate number of participants with missing data for each variable of interest	6
Outcome data	15*	Report numbers of outcome events or summary measures	6
Main results	16	(*a*) Give unadjusted estimates and, if applicable, confounder-adjusted estimates and their precision (eg, 95% confidence interval). Make clear which confounders were adjusted for and why they were included	6
		(*b*) Report category boundaries when continuous variables were categorized	6
		(*c*) If relevant, consider translating estimates of relative risk into absolute risk for a meaningful time period	6
Other analyses	17	Report other analyses done—eg analyses of subgroups and interactions, and sensitivity analyses	6
**Discussion**			
Key results	18	Summarise key results with reference to study objectives	10
Limitations	19	Discuss limitations of the study, taking into account sources of potential bias or imprecision. Discuss both direction and magnitude of any potential bias	13
Interpretation	20	Give a cautious overall interpretation of results considering objectives, limitations, multiplicity of analyses, results from similar studies, and other relevant evidence	13
Generalisability	21	Discuss the generalisability (external validity) of the study results	13
**Other information**			
Funding	22	Give the source of funding and the role of the funders for the present study and, if applicable, for the original study on which the present article is based	14

*Give information separately for cases and controls in case-control studies and, if applicable, for exposed and unexposed groups in cohort and cross-sectional studies.

Note: An Explanation and Elaboration article discusses each checklist item and gives methodological background and published examples of transparent reporting. The STROBE checklist is best used in conjunction with this article (freely available on the Web sites of PLoS Medicine at http://www.plosmedicine.org/, Annals of Internal Medicine at http://www.annals.org/, and Epidemiology at http://www.epidem.com/). Information on the STROBE Initiative is available at www.strobe-statement.org.

For peer review only

Histopathological Profile of Cervical Biopsies in Northern Malawi: A Retrospective Cross-sectional Study

Paul Uchizi Kaseka[1*], Alfred Kayira[1], Chikondi Sharon Chimbatata[1], Master Chisale[1,2,3,] Pocha Kamudumuli[4], Tsung-Shu Joseph Wu[2,5], BalwaniChingatichifwe Mbakaya,[6,7]Frank Watson Sinyiza[1]

[1]Mzuzu Central Hospital, Mzuzu City, Malawi

[2]Luke International, Mzuzu City, Malawi

[3]Mzuzu University, Faculty of Sciences, Technology and Innovations, Biological Sciences, Mzuzu City, Malawi

[4]University of Maryland Global Initiative Corporation, Baltimore, Lilongwe City, Malawi

[5]Pingtung Christian Hospital, Taiwan

[6]University of Livingstonia, Department of Public Health, Malawi

[7]Mzuzu University, Faculty of Health Sciences, Mzuzu City, Malawi

*Corresponding author: kasekapaul2016@gmail.com

ABSTRACT

Objectives: According to the World Health Organization (2014), cervical cancer is the second most common cancer in women globally. More than 85% of the global cervical cancer morbidity and mortality occur in developing countries and the highest risk region is in Eastern and Southern Africa. Malawi has the highest age standardized rate of cervical cancer in the world. This study was carried out to determine the histopathological profile of cervical biopsies in a public tertiary hospital in Mzuzu, northern region of Malawi.

Setting: A public tertiary hospital in Mzuzu, northern region of Malawi

Participants: This was a retrospective study of all cervical biopsy specimen reports received in a public tertiary hospital in northern Malawi over a period of 5 years from July 2013-June 2018. Demographic, clinical and diagnostic data was obtained from original histopathology reports.

Results: A total of 500 cervical biopsy reports were reviewed during the study period. The mean age of the patients was 41.99±12.5. Age ranged from 15 to 80 years. Cervicitis accounted for 46.0% (n=162) of the total nonmalignant lesions seen, followed by cervical intraepithelial neoplasm (CIN), at 24.4% (n=86) and endocervical polyp, at 20.5% (n=72). Squamous cell carcinoma (SCC) accounted for 15.6% (n=78) of the total cervical biopsies studied and 85.7% of all total malignant lesions. Adenocarcinoma and undifferentiated carcinoma were 8.8% and 4.4% respectively of the total malignant diagnosis. All patients with malignant lesions had HIV.

Conclusion: Our study shows that cervicitis and squamous cell carcinoma were most common among nonmalignant and malignant cervical biopsies respectively. Since the frequency of cervical cancer is high, there is need to have well detailed national policies to be put in place to increase detection of pre-invasive lesions in order to reduce the prevalence of cervical cancer.

Key words: Cervical biopsies, cervical cancer, cervical intraepithelial neoplasm, cervicitis, malignant

Strengths and limitation of this study

- The study was able to determine the prevalence and associations of multiple exposures and outcomes.
- This being a retrospective cross-section study, the participants were neither deliberately exposed nor treated; thus, there were no ethical difficulties.
- This study depended on data that were entered into clinical database and not collected in a predesigned proforma as per specific requirements of the study as a result some records were excluded due to missing of certain crucial information.
- Since in retrospective studies, researchers have no control over the exposure of cases versus controls, these unrecognized confounders may have influenced the results.
- The study is a single-hospital-based review and as such inadequate to draw conclusions, but it does shed some light on pathological pattern of cervical cancer in Malawi.

For peer review only - http://bmjopen.bmj.com/site/about/guidelines.xhtml

BACKGROUND

Cervical cancer, after breast cancer, is the second most common cancer in women aged 15 to 44 years and it is the third leading cause of cancer in females worldwide(1). According to the International Agency for Research on Cancer(IARC) estimates, there are 570,000 new cases of cervical cancer annually, resulting into more than 311,000 deaths in 2018 globally(2). Most of the global burden lies in less developed countries, with sub-Saharan Africa (SSA) having the largest age-standardized incidence and mortality rates. Malawi has the highest cervical cancer incidence and mortality in the world with age-standardized rate (ASR) of 75.9 and 49.8 per 100,000 population respectively(3). World Health Organization (WHO) estimates suggest that every year there are at least 3,684 new cases of cervical cancer in Malawi and over 2,314 die from the disease(3).The exact number of cervical cancer morbidity and mortality among Malawian women, is not clear. This could partly be due to unrecorded or underreported cases because of the pathological based cancer registry not being maintained, and also lack of a national system of death certification(4). However, the 2010 National population-based cancer registry indicates that among females, cancer of the cervix was the commonest, accounting for 45.4% of all cases followed by Kaposi's sarcoma (21.1%), cancer of the oesophagus (8.2%), breast cancer (4.6%) and non-Hodgkin lymphoma (4.1%)(5).

The Ministry of Health and Population of the government of Malawi, through the Sexual and Reproductive Health Directorate has implemented a cervical cancer screen-and-treat programme using visual inspection with acetic acid (VIA) approach since 2004, with women between 30-50 years as the main target (6). Women, no younger than 30 years, are offered three (3) free smears, with a 10 years interval in between each smear. Those screened for first time at the age of 55 or more have only one smear if the first smear is normal(6). Cervical screening using VIA is increasingly available in local clinics through the National Cervical Cancer Control Programme(6,7). From clinics, patients are referred to District Hospitals and/or Central Hospitals. At Mzuzu Central Hospital, women with suspected cervical cancer are managed at gynaecology and/or oncology departments. Women with low-grade squamous intraepithelial lesions require

For peer review only - http://bmjopen.bmj.com/site/about/guidelines.xhtml

re-screening within a 12 months' period, whilst those with high-grade lesions are referred for colposcopy.

The standard method for diagnosis of cervical precancerous lesions is histopathological examination of tissue obtained through biopsy guided by colposcopy. When abnormalities are identified, cervical biopsy confirms the diagnosis of cancer(8).This study was carried out to determine the histopathological profile of cervical biopsies in a public tertiary hospital in Mzuzu, northern region of Malawi. The specific objectives were to determine the prevalence of both precancerous and cancerous cervical lesions; characterization of precancerous and cancerous lesions and risk factors of cervical cancer. Understanding the histological pattern of cervical cancer could guide development of focused preventive, care and treatment guidelines to address shortfalls in the care that is provided to cervical cancer patients in Malawi. Ultimately, this could contribute to the global target of 25% reduction of premature mortality from non communicable diseases (NCDs) by the year 2025(9).

MATERIALS AND METHODS

Design, setting and population

This was a retrospective study of all cervical biopsies reports received from Kamuzu Central Hospital/University of North Carolina (KCH/UNC) pathology laboratory in Lilongwe for Mzuzu Central Hospital (MCH), the only public tertiary hospital in the northern region of Malawi. This hospital does not have a functional pathology laboratory and relies on the KCH/UNC pathology laboratory for its services. The hospital is located in the northern part of Malawi catering for a population of about 2,289,780 million people(10)and serving 5 government District Hospitals, 3 Christian Health Association of Malawi (CHAM) hospitals and several private hospitals and clinics.

While some women presented to the gynaecological clinic asymptomatically through screening after VIA indicating a cancer, others presented with symptoms as referrals from a local clinic or other district hospitals from northern region. The most common symptoms were vaginal bleeding (often post coital), vaginal discharge with dysuria,

For peer review only - http://bmjopen.bmj.com/site/about/guidelines.xhtml

abdominal pain, vomiting and weight loss.

Tissue specimens were collected and preserved in 10% buffered formalin solution and then transported to Kamuzu Central Hospital/ University of North Carolina (KCH/UNC) pathology laboratory in Lilongwe. The KCH/UNC laboratory adheres to international quality assurance standards. The flow diagram (Figure 1 below) shows the process involved in coming up with a histopathological diagnosis at KCH/UNC laboratory in Lilongwe.

Within the 5-year period of the study (July 2013 to June 2018), a total of 2294 biopsy reports were received from KCH/UNC Histopathology laboratory. Data extracted included age, year, anatomic site, nature of specimen, clinical diagnosis, histopathological diagnosis, HIV status and whether the specimen was nonmalignant or malignant. Out of 2294 biopsy reports 500 were cervical biopsy reports representing 21.8% of the total biopsy reports received. In this study a total of 500 cervical cancer pathology reports were analyzed. Eleven reports which had missing demographic and clinical data or had inconclusive results were excluded. HIV status was defined as whether the patient was HIV sero reactive (positive), negative or not tested (unknown) when the biopsy was being taken. HPV status was defined as the patient sample being positive or negative upon histopathology examination.

Patient and public involvement

No patient involved

Data analysis

Data were entered in Microsoft excel 2016, validated and cleaned before importing into Stata, version 13.0 (Stata Corp. LP, College Station, TX, United States of America) for analysis. Descriptive analyses were performed to summarize patients' sociodemographic and clinical characteristics. A multivariate logistic regression was used to estimate the magnitude of the association between predictor variables (age and HIV status) and cancerat 95% confidence level.

For peer review only - http://bmjopen.bmj.com/site/about/guidelines.xhtml

Ethical clearance

This study was approved by National Health Science Research Committee (NHSRC) as part of the main study "Pathological profile of malignancies in northern Malawi-A retrospective study at Mzuzu Central Hospital"number 19/05/2316.Both the MCH Research and Publication Committee and the MCH Laboratory department consents were obtained for the study. The need for informed consent was exempted from the institutional review board due to the nature of the study, which involved a retrospective analysis of routinely collected data.

RESULTS

Within the 5-year period of the study (July 2013 to June 2018), a total of 500 biopsy reports were received from KCH/ UNC Pathology laboratory. The mean age of patients included in this study was 41.99 ± 12.5. The age range was 15-80 years. Most of the cervical biopsies were from patients in the 31-40 age group (34.8% n=174). Twelve percent of the cervical biopsies were from HIV positive patients and 51% of the results had unknown HIV result (Table 1).

Table 1. Demographic profile of cervical biopsies

Parameter		Biopsies analysed	
		Frequency	Percentage (%)
Age(Mean)years	20 and below (17)	3	0.6
	21-30(27)	57	11.4
	31-40(36)	174	34.8
	41-50(45)	132	26.4
	51-60(54)	84	16.8
	61-70(65)	29	5.8
	Above 70(75)	7	1.4
	Missing	14	2.8

For peer review only - http://bmjopen.bmj.com/site/about/guidelines.xhtml

HIV status	Positive	61	12.2
	Negative	184	36.8
	Unknown (not documented)	255	51.0

Of all the cervical biopsies studied, 91 (18.2%) were malignant. Squamous cell carcinoma (SCC) accounted for 85.7% of all malignant lesions (Table 2). Cervicitis accounted for 46.0% (n=162) of the total nonmalignant lesions seen. Ten percent of cervicitis cases and 30.2% of CIN cases had HPV respectively (Table 2).

Table 2. Histopathological diagnosis of cervical biopsies

Parameter	Frequency	Within group percentage(%)	Percentage(%)total
**Normal cervical tissue**	**46**		**9.2**
**Nonmalignant lesions**	**352**		**70.4**
Cervicitis*	162	46.0	32.4
Cervical intraepithelial neoplasm (CIN)*	86	24.4	17.2
Endocervical polyp	72	20.5	14.4
Carcinoma in situ	4	1.1	0.8
Nabothian cyst	4	1.1	0.8
Schistosomiasis	5	1.4	1.0
Condyloma (warts)	11	3.1	2.2
Others	8	2.3	1.6
**Malignant**	**91**		**18.2**
Squamous cell carcinoma	78	85.7	15.6
Adenocarcinoma	8	8.8	1.6
Undifferentiated	4	4.4	0.8
Carcinosaroma	1	1.1	0.2
**Inconclusive result**	**11**		**2.2**

For peer review only - http://bmjopen.bmj.com/site/about/guidelines.xhtml

**Total**	**500**	**100**

*Cervicitis with HPV= 10% (16/162) *CIN with HPV= 30.2% (26/86)

*CIN 1 = 34.9% (30/86), CIN 2 = 22.1% (19/86), CIN 3 = 43.0% (37/86)

The chances of developing cervical cancer increased with advancing age, with the risk becoming more prominent after age 60 years. Women aged 61-70 years were 6.2 times (OR = 6.2, 95% CI: 2.1 - 18.4)more likely to develop cervical cancer than those in the 21-30 years' age category. The odds of getting cancer were higher in women living with HIV. Women with HIV were more than twice (OR = 2.3, 95% CI: 1.1 - 4.9) as likely to have cervical cancer as those who were HIV negative (Table 3).

For peer review only

Table 3: Association between cancer, age and HIV status

		Cancer (%)	Non cancer (%)	Unadjusted odds ratio (95% CI)†	Adjusted odds ratio (95% CI)†
Age (years)	20 and below	0 (0.0)	2 (100.0)		
	21 - 30 (reference)	7 (12.3)	50 (87.2)	-	
	31 - 40	25 (14.6)	146 (85.4)	1.2 (0.5 -3.0)	1.2 (0.5- 2.9)
	41 - 50	23 (17.7)	107 (82.3)	1.5 (0.6 - 3.8)	1.5 (0.6 - 3.8)
	51 - 60	18 (22.2)	63 (77.8)	2.0 (0.8 - 5.3)	2.2 (0.8 - 5.7)
	61 - 70	13 (44.8)	16 (55.2)	5.8 (2.0 - 17.0)	6.2 (2.1 - 18.4)
	Above 70	1 (20.0)	4 (80.0)	1.8 (0.2 - 18.3)	1.9 (0.2 - 19.9)
HIV status	Negative (reference)	26 (14.4)	155 (85.6)	-	
	Positive	15 (24.6)	46 (75.4)	1.9 (1.0 - 4.0)	2.3 (1.1 - 4.9)
	Unknown	50 (20.2)	197 (79.8)		

Fourteen patients (4 cancer cases and 10 non cancer cases) had no data on age and were excluded from this analysis

†Binary logistic regression

For peer review only - http://bmjopen.bmj.com/site/about/guidelines.xhtml

DISCUSSION

To the best of our knowledge, this is the first study conducted to determine the histopathological profile of cervical biopsies in a public tertiary hospital in Mzuzu, northern region of Malawi. This study found more benign conditions (70.4% n=352) than neoplastic malignant conditions (18.2% n=91). This is in contrast with other studies undertaken in developing countries which reported more neoplastic malignant conditions than benign conditions(8,9).

Our study found that cervicitis, an inflammatory disease comprising acute cervicitis and chronic non-specific cervicitis was the most common nonmalignant condition. It accounted for 46% of all the nonmalignant tumours and 32.4% of all cervical biopsies received in this study. This was consistent to a study conducted in Nigeria which had 37.5% cervicitis of all cervical biopsies included in that study(11). Most cases of cervicitis are often due to non-specific causes or infective agents(12). Cervicitis has been highly reported in previously studies inother countries(12,13).

In this study CIN, a premalignant lesion ranked second in nonmalignant lesions accounting for 24% of all nonmalignant lesions and 17.2% of all cervical biopsies with the prevalence of CIN I, CIN II, CIN III of 34.9%, 22.1% and 43% respectively. This finding is comparable to a study conducted in Nigeria which reported CIN constituting 15% of all cervical biopsies reviewed(13). In that study however, there was a reduction in the prevalence of CIN from low grade CIN to high grade CIN(13). In our study, the high grade CIN (CIN III) was the most common. This finding indicates that invasive cervical cancer progresses from advanced stages of precancerous lesions. This shows that there is need to create community awareness and strengthen early cervical cancer screening for Malawi to have better outcomes.

In the current study 10% (n=16) of all the cervicitis cases and 30.2% (n=26) of all the CIN biopsies had HPV. This finding suggests that there is high rate of HPV infections, the causative agent of CIN in the younger age group(13). Unfortunately, the data on population-based, age specific prevalence of HPV are not available in Malawi. However, WHO estimates that the overall HPV prevalence in Malawi is about 34%(7). HPV

For peer review only - http://bmjopen.bmj.com/site/about/guidelines.xhtml

infection and precancerous lesions are usually difficult to notice and develop into full blown cancer before women realize the need to seek medical care.

The rate of malignant lesions (tumours) in our study (18.2%) is comparable to 16.2% and 12.2% in studies conducted in Benin City(14), and in Enugu, in Nigeria(15) respectively. Studies done in South Africa, Saudi Arabia, India and United States, with malignant lesions at 2.42%, 4.95%, 5.5% and 5.0% respectively, are all at variance with the current study which explains that early cervical cancer screening helps to reduce the prevalence of cervical cancer(16–19).

Among the malignant tumours, SCC was the most common histological type of cervical cancer in our study. SCC accounted for 85.7% of malignant lesions cervical cancer and was also the most common diagnosis, at 15.6% of all cervical biopsies in this study. This is consistent with findings from other studies conducted worldwide (20–22). As Faduyile et al. observed the high rate of SCC in Malawi and Africa could reflect the low uptake of VIA and Pap smear test which are capable of identifying dysplastic conditions before transformation to malignancy(11). This shows that there is need for Malawi to have well organized cervical cancer screening and Pap smear test to reduce the prevalence of SCC(2).

In our study, adenocarcinorma and poorly differentiated carcinorma were 8.8% and 4.4% respectively of the total malignant diagnosis. The prevalence of adenocarcinoma and poorly differentiated carcinoma observed in this study is in contrast to other studies done in Nigeria where one study found 5.8% and 2.0% of the total malignant lesions diagnosis and another found 6.0% and 1.0% respectively(13,23). The high prevalence found in this study confirms a previous study findings by Chanza et al.(24). Chanza et al. reported that Malawian women delay to seek medical attention due to limited knowledge on symptoms and signs, limited financial resources, limited accessibility and unavailability of cancer screening facilities hence late diagnosis. These results show that early cervical cancer screening, increased awareness, better health care facilities, accessibility, improved histopathological confirmatory diagnosis and early treatment by surgeons, may reduce the cervical cancer burden in Malawi.

For peer review only - http://bmjopen.bmj.com/site/about/guidelines.xhtml

In the current study it has been observed that the diagnosis of cervical cancer was significantly associated with the age of the patients. The chances of developing cervical cancer increased with advancing age, with the risk becoming more prominent after age 60 years. Women aged 61-70 years were 6.2 times (OR = 6.2, 95% CI: 2.1 - 18.4) more likely to develop cervical cancer than those in the 21-30 years age category(Table 3). It is important to note that high quality screening programs are important to prevent cervical cancer among unvaccinated older women(25). None of the women in our study had received any HPV vaccine in their lifetime as the first round of the mass HPV vaccine in this country was administered in January 2019 mainly in schools, targeting 9-13 year old girls who had not yet become sexually active and the second round was given in January 2020. In Malawi, the integration of HPV vaccine programs with adequate screening programs in older women (aged 30-49 years) has the potential to reduce the burden of cervical cancer.

Malawi's HIV prevalence is one of the highest in the world, with 10.6% of adult population (aged 15-64) living with HIV(26). With such a high HIV prevalence in Malawi, there is an increased risk of AIDS-defining cancers including cervical cancer. In this study the probability of getting cancer were higher in women living with HIV. Women with HIV were more than twice (OR = 2.3, 95% CI: 1.1 - 4.9)as likely to have cervical cancer as those who were HIV negative (Table 3).This could be attributed to the reason that HIV-infected women are more likely than HIV-uninfected women to have incident and persistent HPV cervical infections(27).A twelve monthlycervical cancer screening, increased availability of dolutegravir (DTG) based antiretroviral treatment (ART) to HIV infected women now being provided in the country, routine viral load checks (at 6 months, 12 months and every 12 months since initiation) and timely switch to second line or third line regimens to ensure viral load suppression will eventually have an impact on benign lesions progressing to invasive cervical cancer(28). However, in this study almost half (52.74%, n=48) of all the women diagnosed with cervical cancer had an unknown HIV status. This calls for scaling up of HIV testing in cancer screening settings for early diagnosis and ART referral and further research is warranted on barriers among HIV-infected women to seeking cancer screening services despite already being in the health care system.

For peer review only - http://bmjopen.bmj.com/site/about/guidelines.xhtml

LIMITATIONS

This study used available programme health facility data and histopathological reports on cervical cancer. The use of health facility data has its own limitations, such as incompleteness and bias in the sense that information is obtained only from people who came to the facility and underwent biopsy, leaving out those that did not seek medical care and or were not biopsied and therefore cannot be generalized to the general population.

The other limitation of this study is that it is a single-hospital-based review and as such inadequate to draw conclusions, but it does shed some light on pathological pattern of cervical cancer in Malawi.

Finally, this is a retrospective study so we could not be able to extract details for example in cases where tumours were diagnosed by screening or symptoms, presence or absence of the patient co-morbidities. Nevertheless, the comprehensive histopathological pattern of cervical cancer demonstrated by this study provides evidence that could be used to inform policies, strategies and intervention for prevention of cancer in Malawi.

CONCLUSION

The SCC was the commonest malignant condition and cervicitis and CIN were the most common non malignant conditions in all the women studied. Since the frequency of cervical cancer is high, there is need for well detailed national policies to be put in place to increase detection of pre-invasive lesions, which in turn will decrease the frequency of cervical cancer in the country. The presence of chronic non-specific cervicitis in women of reproductive age is infective in origin with its attending sequelae. Intensifying screening programmes among women and provision of long term ART to the HIV infected may offer an opportunity for appropriate interventions to reduce morbidity, mortality and reduce complications among these women.

Declarations

i. Ethical approval and consent to participant

For peer review only - http://bmjopen.bmj.com/site/about/guidelines.xhtml
For peer review only

This study was approved by National Health Science Research Committee as part of the main study "Pathological profile of malignancies in northern Malawi-A retrospective study at Mzuzu Central Hospital" number 19/05/2316.Both the Mzuzu Central Hospital Research and Publication Committee and the Mzuzu Central Hospital Laboratory department consents were obtained for the study. The need for informed consent was exempted from institutional review board due to the nature of the study, which involved a retrospective analysis of routinely collected data.

ii. **Consent for publication:** Not applicable

iii. **Availability of data and materials**

The datasets used and/or analyzed during the current study are available from the corresponding author on reasonable request. Email: kasekapaul2016@gmail.com

ORCID: 0000-0002-6651-8000

iv. **Competing interests:** The authors declare that they have no competing interest

v. **Funding**

The study's data collection, analysis, interpretation of data and manuscript writing was funded by Pingtung Christian Hospital, Taiwan through Luke International Norway (LIN), Malawi, Grant Number: PS-IR-108001

vi. **Authors contributions**

PUK and FWS conceived and designed the study. AK, CC, PK, TJW, MC and BCM contributed to development of the study protocol and supervised data collection and entry. AK analyzed the data and PUK drafted the manuscript. All authors read and approved the final manuscript.

vii. **Acknowledgements**

The authors are sincerely grateful to all the data collectors and the Laboratory manager for providing the data. The Pingtung Christian Hospital through Luke International Norway (LIN) is also appreciated for financial support.

For peer review only - http://bmjopen.bmj.com/site/about/guidelines.xhtml

REFERENCES

1. Bruni L, Albero G, Serrano B et al. ICO/IARC Information Centre on HPVand Cancer (HPV Information Centre). Human Papillomavirus and Related Diseases in the World. Summary Report 17 June 2019.www.hpvcentre.net [Accessed 15 October 2019].
2. Bray F, Ferlay J, Soerjomataram I et al. Global cancer statistics 2018: GLOBOCAN estimates of incidence and mortality worldwide for 36 cancers in 185 countries. A Cancer J Clin. 2018;68(6):394–424. DOI: 10.3322/caac.21492.
3. Ferlay J, Soerjomataram I, Ervik M et al. IARC Publications Website - GLOBOCAN 2012: Estimated Cancer Incidence, Mortality and Prevalence Worldwide in 2012 v1.0 [Internet]. WHO, International Research on Cancer. 2012ISBN-13 (Database) 978-92-832-2447-1 [cited 2019 Nov 18]. Available from: https://publications.iarc.fr/Databases/Iarc-Cancerbases/GLOBOCAN-2012-Estimated-Cancer-Incidence-Mortality-And-Prevalence-Worldwide-In-2012-V1.0-2012
4. Rudd P, Gorman D, Meja S et al. Cervical cancer in southern Malawi: A prospective analysis of presentation, management and outcomes. Malawi Med J. 2017;29(2):124–9.PMID: 28955419.
5. Msyamboza KP, Ngwira B, D. The burden of selected chronic non-communicable diseases and their risk factors in malawi: Nationwide steps survey. PLoS One. 2011;6(5):611.DOI.org/10.1371/journal.pone.0020316https://doi.org/10.1371/journal.pone.0020316.
6. Msyamboza KP, Phiri T, Sichali W, Kwenda W, Kachale F. Cervical cancer screening uptake and challenges in Malawi from 2011 to 2015: Retrospective cohort study. BMC Public Health [Internet]. 2016;16(1):1–6. Available from: http://dx.doi.org/10.1186/s12889-016-3530-y
7. Malawi Ministry of Health and Population, UNFPA. Malawi National cervical cancer control Strategy 2016-2020 [Internet]. Unfpa. 2017. Available from: http://www.embase.com/search/results?subaction=viewrecord&from=export&id=L71343074%5Cnhttp://dx.doi.org/10.1158/1538-7445.AM2013-ADV01%5Cnhttp://fq5np7af6j.search.serialssolutions.com?sid=EMBASE&issn=00085472&id=doi:10.1158/1538-7445.AM2013-ADV01&atitle=N
8. Itano J, Taoka KN. Core curriculum for oncology nursing. Elsevier Saunders; 2005. 970 p.
9. Noncommunicable diseases country profiles 2018. Geneva: World Health Organization; 2018. Licence: CC BY-NC-SA 3.0 IGO.
10. National Statistical O. 2018 Malawi Population and Housing Census Main Report [Internet]. 2019. Available from: http://10.150.72.21:6510/www.nsomalawi.mw/images/stories/data_on_line/demography/census_2018/2018 Malawi Population and Housing Census Main Report.pdf
11. Omoniyi-esan OG, Osasan SA, Ojo OS. Non-neoplastic diseases of the cervix in Nigerians : A histopathological study. African Heal Sci Vol. 2006;6(2):76–80.PMCID: PMC1831974 PMID: 16916295.
12. Nwachokor FN, Forae GD. Morphological spectrum of non - neoplastic lesions of

For peer review only - http://bmjopen.bmj.com/site/about/guidelines.xhtml

the uterine cervix in Warri , South - South , Nigeria. 2013;16(4):429–32.DOI: 10.4103/1119-3077.116883.
13. Faduyile FA, Soyemi SS, Wright KO et al. Histopathological study of surgical cervical biopsies in Lagos ,. Trop J Obstet Gynaecol. 2017;34(2):124–8.DOI: 10.4103/TJOG.TJOG_28_17.
14. Obaseki D, Nwafor C. Cervical Cancer Screening In Benin City, South-South Nigeria [Internet]. Vol. 5, IOSR Journal of Dental and Medical Sciences (IOSR-JDMS) e-ISSN. 2013 [cited 2019 Nov 18]. e-ISSN: 2279-0853, p-ISSN: 2279-0861. www.iosrjournals.org. Available from: www.iosrjournals.org
15. Chukwuali LI, Onuigho WIB, Mgbor NC. Cervical cancer screening in Enugu, Nigeria. Trop J Obs Gynaecol. 2003;20:109–12.DOI: 10.4314/tjog.v20i2.14412.
16. Fonn S, Bloch B, Mabina M et al. Prevalence of pre-cancerous leisons and cervical cancer in South Africa-A Multicentre study. SAMJ. 2002;92(2):148–56. PMID: 11894653.
17. Balaha MH, Moghannum MS Al, Ghowinem NA et al. Cytological pattern of cervical Papanicolaou smear in eastern region of Saudi Arabia. J Cytol. 2011;28(4):173–177. DOI: 10.4103/0970-9371.86343

. 18. Patel MM, Pandya AN, Modi J. Cervical pap smear study and its utility in cancer screening, to specify the strategy for cervical cancer control. Natl J COMMUNITY Med. 2011;2(1):49–51. ISSN: 0976 3325.
19. Insinga RP, Glass AG, Rush BB. Diagnoses and outcomes in cervical cancer screening: A population-based study. Am J Obstet Gynecol. 2004 Jul;191(1):105–13. https://doi.org/10.1016/j.ajog.2004.01.043.
20. Okoye C. Histopathological pattern of cervical cancer in Benin City, Nigeria. J Med Investig Pract. 2014 Oct 1;9(4):147–147. link.gale.com/apps/doc/A414542015/AONE?u=nysl_ca_ind&sid=googleScholar&xid=7c182fa6. Accessed 18 Aug. 2021. Gale Document Number: GALE|A414542015.

21. Pathak T, Pun C, Shrestha S et al. Incidence trends and histopathological pattern of cervical malignancies at BP koirala memorial centre hospital, Nepal. J Pathol Nepal. 2013;3:386–9. DOI: https://doi.org/10.3126/jpn.v3i5.7863.
22. Badar F, Anwar N, Meerza F et al. Cervical Carcinoma in a Muslim Community. Asian Pacific J Cancer Prev. 2007;8:24–6. PMID: 17477766.
23. Olu-Eddo AN, Ekanem VJ, Umannah I et al. A 20 year histopathological study of cancer of the cervix in Nigerians. Nig Q J Hosp Med. 2011 Apr;21(2):149–53. PMID: 21913514.
24. Chadza E, Chirwa E, Maluwa A et al. Factors that contribute to delay in seeking cervical cancer diagnosis and treatment among women in Malawi. 2012;4(11):1015–22. DOI: 10.4236/health.2012.411155.
25. Soares GR, Vieira R da R, Pellizzer EP et al. Indications for the HPV vaccine in adolescents: A review of the literature. J Infect Public Health [Internet]. . 2015;8(2):105–16. Available from: http://dx.doi.org/10.1016/j.jiph.2014.08.011. http://dx.doi.org/10.1016/j.jiph.2014.08.011.
26. Malawi Population-based HIV Impact Assessment Survey - MPHIA 2015 - 2016

For peer review only

[Internet]. Available from: https://phia.icap.columbia.edu/wp-content/uploads/2019/08/MPHIA-Final-Report_web.pdf
27. Abraham AG, Strickler HD, Jing Y et al. Invasive cervical cancer risk among HIV-infected women: A North American multi-cohort collaboration prospective study. J Acquir Immune Defic Syndr. 2013;62(4):405–13. doi: 10.1097/QAI.0b013e31828177d7.
28. Ministry of Health and P. Malawi Guidelines for Clinical Management of HIV in Children and Adults (Third Edition, 2016) [Internet]. Lilongwe; 2016 [cited 2019 Nov 19]. Available from: www.hiv.health.gov.mw

For peer review only

For peer review only - http://bmjopen.bmj.com/site/about/guidelines.xhtml

APPENDIX 1

Figure 1. Process involved in specimen processing at KCH/UNC laboratory

For peer review only

For peer review only - http://bmjopen.bmj.com/site/about/guidelines.xhtml

BMJ Open

Histopathological Profile of Cervical Biopsies in Northern Malawi: A Retrospective Cross-sectional Study

Journal:	*BMJ Open*
Manuscript ID	bmjopen-2020-048283.R2
Article Type:	Original research
Date Submitted by the Author:	13-Jan-2022
Complete List of Authors:	Kaseka, Paul; Mzuzu Central Hospital,<br>Kayira, Alfred; Mzuzu Central Hospital<br>Chimbatata, Chikondi; Mzuzu Central Hospital<br>Chisale, Master; Mzuzu University, Biological Sciences; Mzuzu University<br>Kamudumuli, Pocha; University of Maryland and Global Initiative Company<br>Wu, Tsung-Shu; Ping Tung Christian Hospital<br>Mbakaya, Balwani; University of Livingstonia<br>Sinyiza, Frank; Mzuzu Central Hospital
**Primary Subject Heading**:	Oncology
Secondary Subject Heading:	Obstetrics and gynaecology, Pathology
Keywords:	ONCOLOGY, OBSTETRICS, Histopathology < PATHOLOGY, Adult oncology < ONCOLOGY, Gynaecological oncology < GYNAECOLOGY

SCHOLARONE™
Manuscripts

I, the Submitting Author has the right to grant and does grant on behalf of all authors of the Work (as defined in the below author licence), an exclusive licence and/or a non-exclusive licence for contributions from authors who are: i) UK Crown employees; ii) where BMJ has agreed a CC-BY licence shall apply, and/or iii) in accordance with the terms applicable for US Federal Government officers or employees acting as part of their official duties; on a worldwide, perpetual, irrevocable, royalty-free basis to BMJ Publishing Group Ltd ("BMJ") its licensees and where the relevant Journal is co-owned by BMJ to the co-owners of the Journal, to publish the Work in this journal and any other BMJ products and to exploit all rights, as set out in our licence.

The Submitting Author accepts and understands that any supply made under these terms is made by BMJ to the Submitting Author unless you are acting as an employee on behalf of your employer or a postgraduate student of an affiliated institution which is paying any applicable article publishing charge ("APC") for Open Access articles. Where the Submitting Author wishes to make the Work available on an Open Access basis (and intends to pay the relevant APC), the terms of reuse of such Open Access shall be governed by a Creative Commons licence – details of these licences and which Creative Commons licence will apply to this Work are set out in our licence referred to above.

Other than as permitted in any relevant BMJ Author's Self Archiving Policies, I confirm this Work has not been accepted for publication elsewhere, is not being considered for publication elsewhere and does not duplicate material already published. I confirm all authors consent to publication of this Work and authorise the granting of this licence.

For peer review only

Histopathological Profile of Cervical Biopsies in Northern Malawi: A Retrospective Cross-sectional Study

Paul Uchizi Kaseka[1*], Alfred Kayira[1], Chikondi Sharon Chimbatata[1], Master Chisale[1,2,3,] Pocha Kamudumuli[4], Tsung-Shu Joseph Wu[2,5], BalwaniChingatichifwe Mbakaya,[6,7]Frank Watson Sinyiza[1]

[1]Mzuzu Central Hospital, Mzuzu City, Malawi

[2]Luke International, Mzuzu City, Malawi

[3]Mzuzu University, Faculty of Sciences, Technology and Innovations, Biological Sciences, Mzuzu City, Malawi

[4]University of Maryland Global Initiative Corporation, Baltimore, Lilongwe City, Malawi

[5]Pingtung Christian Hospital, Taiwan

[6]University of Livingstonia, Department of Public Health, Malawi

[7]Mzuzu University, Faculty of Health Sciences, Mzuzu City, Malawi

*Corresponding author: kasekapaul2016@gmail.com

For peer review only

ABSTRACT

Objectives: According to the World Health Organization (2014), cervical cancer is the second most common cancer in women globally. More than 85% of the global cervical cancer morbidity and mortality occur in developing countries and the highest risk region is in Eastern and Southern Africa. Malawi has the highest age standardized rate of cervical cancer in the world. This study was carried out to determine the histopathological profile of cervical biopsies in a public tertiary hospital in Mzuzu, northern region of Malawi.

Setting: A public tertiary hospital in Mzuzu, northern region of Malawi

Participants: This was a retrospective study of all cervical biopsy specimen reports received in a public tertiary hospital in northern Malawi over a period of 5 years from July 2013-June 2018. Demographic, clinical and diagnostic data was obtained from original histopathology reports.

For peer review only - http://bmjopen.bmj.com/site/about/guidelines.xhtml

Results: A total of 500 cervical biopsy reports were reviewed during the study period. The mean age of the patients was 41.99±12.5. Age ranged from 15 to 80 years. Cervicitis accounted for 46.0% (n=162) of the total nonmalignant lesions seen, followed by cervical intraepithelial neoplasm (CIN), at 24.4% (n=86) and endocervical polyp, at 20.5% (n=72). Squamous cell carcinoma (SCC) accounted for 15.6% (n=78) of the total cervical biopsies studied and 85.7% of all total malignant lesions. Adenocarcinoma and undifferentiated carcinoma were 8.8% and 4.4% respectively of the total malignant diagnosis. All patients with malignant lesions had HIV.

Conclusion: Our study shows that cervicitis and squamous cell carcinoma were most common among nonmalignant and malignant cervical biopsies respectively. Since the frequency of cervical cancer is high, there is need to have well detailed national policies to be put in place to increase detection of pre-invasive lesions in order to reduce the prevalence of cervical cancer.

Key words: Cervical biopsies, cervical cancer, cervical intraepithelial neoplasm, cervicitis, malignant

Strengths and limitation of this study

- The study was able to determine the prevalence and associations of multiple exposures and outcomes.
- This being a retrospective cross-section study, the participants were neither deliberately exposed nor treated; thus, there were no ethical difficulties.
- This study depended on data that were entered into clinical database and not collected in a predesigned proforma as per specific requirements of the study as a result some records were excluded due to missing of certain crucial information.
- Since in retrospective studies, researchers have no control over the exposure of cases versus controls, these unrecognized confounders may have influenced the results.
- The study is a single-hospital-based review and as such inadequate to draw conclusions, but it does shed some light on pathological pattern of cervical cancer in Malawi.

For peer review only

BACKGROUND

Cervical cancer, after breast cancer, is the second most common cancer in women aged 15 to 44 years and it is the third leading cause of cancer in females worldwide(1). According to the International Agency for Research on Cancer(IARC) estimates, there are 570,000 new cases of cervical cancer annually, resulting into more than 311,000 deaths in 2018 globally(2). Most of the global burden lies in less developed countries, with sub-Saharan Africa (SSA) having the largest age-standardized incidence and mortality rates. Malawi has the highest cervical cancer incidence and mortality in the world with age-standardized rate (ASR) of 75.9 and 49.8 per 100,000 population respectively(3). World Health Organization (WHO) estimates suggest that every year there are at least 3,684 new cases of cervical cancer in Malawi and over 2,314 die from the disease(3).The exact number of cervical cancer morbidity and mortality among Malawian women, is not clear. This could partly be due to unrecorded or underreported cases because of the pathological based cancer registry not being maintained, and also lack of a national system of death certification(4). However, the 2010 National population-based cancer registry indicates that among females, cancer of the cervix was the commonest, accounting for 45.4% of all cases followed by Kaposi's sarcoma (21.1%), cancer of the oesophagus (8.2%), breast cancer (4.6%) and non-Hodgkin lymphoma (4.1%)(5).

The Ministry of Health and Population of the government of Malawi, through the Sexual and Reproductive Health Directorate has implemented a cervical cancer screen-and-treat programme using visual inspection with acetic acid (VIA) approach since 2004, with women between 30-50 years as the main target (6). Women, no younger than 30 years, are offered three (3) free smears, with a 10 years interval in between each smear. Those screened for first time at the age of 55 or more have only one smear if the first smear is normal(6). Cervical screening using VIA is increasingly available in local clinics through the National Cervical Cancer Control Programme(6,7). From clinics, patients are referred to District Hospitals and/or Central Hospitals. At Mzuzu Central Hospital, women with suspected cervical cancer are managed at gynaecology and/or oncology departments. Women with low-grade squamous intraepithelial lesions require

For peer review only - http://bmjopen.bmj.com/site/about/guidelines.xhtml

re-screening within a 12 months' period, whilst those with high-grade lesions are referred for colposcopy.

The standard method for diagnosis of cervical precancerous lesions is histopathological examination of tissue obtained through biopsy guided by colposcopy. When abnormalities are identified, cervical biopsy confirms the diagnosis of cancer(8).This study was carried out to determine the histopathological profile of cervical biopsies in a public tertiary hospital in Mzuzu, northern region of Malawi. The specific objectives were to determine the prevalence of both precancerous and cancerous cervical lesions; characterization of precancerous and cancerous lesions and risk factors of cervical cancer. Understanding the histological pattern of cervical cancer could guide development of focused preventive, care and treatment guidelines to address shortfalls in the care that is provided to cervical cancer patients in Malawi. Ultimately, this could contribute to the global target of 25% reduction of premature mortality from non communicable diseases (NCDs) by the year 2025(9).

MATERIALS AND METHODS

Design, setting and population

This was a retrospective study of all cervical biopsies reports received from Kamuzu Central Hospital/University of North Carolina (KCH/UNC) pathology laboratory in Lilongwe for Mzuzu Central Hospital (MCH), the only public tertiary hospital in the northern region of Malawi. This hospital does not have a functional pathology laboratory and relies on the KCH/UNC pathology laboratory for its services. The hospital is located in the northern part of Malawi catering for a population of about 2,289,780 million people(10)and serving 5 government District Hospitals, 3 Christian Health Association of Malawi (CHAM) hospitals and several private hospitals and clinics.

While some women presented to the gynaecological clinic asymptomatically through screening after VIA indicating a cancer, others presented with symptoms as referrals from a local clinic or other district hospitals from northern region. The most common symptoms were vaginal bleeding (often post coital), vaginal discharge with dysuria,

For peer review only - http://bmjopen.bmj.com/site/about/guidelines.xhtml

abdominal pain, vomiting and weight loss.

Tissue specimens were collected and preserved in 10% buffered formalin solution and then transported to Kamuzu Central Hospital/ University of North Carolina (KCH/UNC) pathology laboratory in Lilongwe. The KCH/UNC laboratory adheres to international quality assurance standards. The flow diagram (see Figure 1 attached) shows the process involved in coming up with a histopathological diagnosis at KCH/UNC laboratory in Lilongwe.

Within the 5-year period of the study (July 2013 to June 2018), a total of 2294 biopsy reports were received from KCH/UNC Histopathology laboratory. Data extracted included age, year, anatomic site, nature of specimen, clinical diagnosis, histopathological diagnosis, HIV status and whether the specimen was nonmalignant or malignant. Out of 2294 biopsy reports 500 were cervical biopsy reports representing 21.8% of the total biopsy reports received. In this study a total of 500 cervical cancer pathology reports were analyzed. Eleven reports which had missing demographic and clinical data or had inconclusive results were excluded. HIV status was defined as whether the patient was HIV sero reactive (positive), negative or not tested (unknown) when the biopsy was being taken. HPV status was defined as the patient sample being positive or negative upon histopathology examination. HPV was diagnosed histologically through observation of dysplastic changes in the superficial cervical epithelium that are consistent with HPV infection. These changes include koilocytosis and chronic inflammation. Histologically, koilocytosis is characterized by perinuclear cavitation, enlarged nucleus with coarse chromatin making it stain dark (hyperchromasia) with Lugol’s iodine solution, irregular nuclear membranes and a rim of condensed cytoplasm around the perinuclear cavitation which gives the cells a ‘halo’ or cleared-out appearance around the dysplastic nucleus. Chronic inflammation on the other hand is characterized by infiltration of inflammatory cells (lymphocytes) into the cervical tissue.

Patient and public involvement

No patient involved

Data analysis

Data were entered in Microsoft excel 2016, validated and cleaned before importing into Stata, version 13.0 (Stata Corp. LP, College Station, TX, United States of America) for

For peer review only - http://bmjopen.bmj.com/site/about/guidelines.xhtml

analysis. Descriptive analyses were performed to summarize patients' sociodemographic and clinical characteristics. A multivariate logistic regression was used to estimate the magnitude of the association between predictor variables (age and HIV status) and cancerat 95% confidence level.

Ethical clearance

This study was approved by National Health Science Research Committee (NHSRC) as part of the main study "Pathological profile of malignancies in northern Malawi-A retrospective study at Mzuzu Central Hospital"number 19/05/2316.Both the MCH Research and Publication Committee and the MCH Laboratory department consents were obtained for the study. The need for informed consent was exempted from the institutional review board due to the nature of the study, which involved a retrospective analysis of routinely collected data.

RESULTS

Within the 5-year period of the study (July 2013 to June 2018), a total of 500 biopsy reports were received from KCH/ UNC Pathology laboratory. The mean age of patients included in this study was 41.99 ± 12.5. The age range was 15-80 years. Most of the cervical biopsies were from patients in the 31-40 age group (34.8% n=174). Twelve percent of the cervical biopsies were from HIV positive patients and 51% of the results had unknown HIV result (Table 1).

Table 1. Demographic profile of cervical biopsies

Parameter		Biopsies analysed	
		Frequency	Percentage (%)
Age(Mean)years	20 and below (17)	3	0.6
	21-30(27)	57	11.4
	31-40(36)	174	34.8

For peer review only - http://bmjopen.bmj.com/site/about/guidelines.xhtml

	41-50(45)	132	26.4
	51-60(54)	84	16.8
	61-70(65)	29	5.8
	Above 70(75)	7	1.4
	Missing	14	2.8
HIV status	Positive	61	12.2
	Negative	184	36.8
	Unknown (not documented)	255	51.0

Of all the cervical biopsies studied, 91 (18.2%) were malignant. Squamous cell carcinoma (SCC) accounted for 85.7% of all malignant lesions (Table 2). Cervicitis accounted for 46.0% (n=162) of the total nonmalignant lesions seen. Ten percent of cervicitis cases and 30.2% of CIN cases had HPV respectively (Table 2).

Table 2. Histopathological diagnosis of cervical biopsies

Parameter	Frequency	Within group percentage(%)	Percentage(%)total
**Normal cervical tissue**	**46**		**9.2**
**Nonmalignant lesions**	**352**		**70.4**
Cervicitis*	162	46.0	32.4
Cervical intraepithelial neoplasm (CIN)*	86	24.4	17.2
Endocervical polyp	72	20.5	14.4
Carcinoma in situ	4	1.1	0.8
Nabothian cyst	4	1.1	0.8
Schistosomiasis	5	1.4	1.0
Condyloma (warts)	11	3.1	2.2

For peer review only - http://bmjopen.bmj.com/site/about/guidelines.xhtml

Others	8	2.3	1.6
**Malignant**	**91**		**18.2**
Squamous cell carcinoma	78	85.7	15.6
Adenocarcinoma	8	8.8	1.6
Undifferentiated	4	4.4	0.8
Carcinosaroma	1	1.1	0.2
**Inconclusive result**	**11**		**2.2**
**Total**	**500**		**100**

*Cervicitis with HPV= 10% (16/162) *CIN with HPV= 30.2% (26/86)

*CIN 1 = 34.9% (30/86), CIN 2 = 22.1% (19/86), CIN 3 = 43.0% (37/86)

The chances of developing cervical cancer increased with advancing age, with the risk becoming more prominent after age 60 years. Women aged 61-70 years were 6.2 times (OR = 6.2, 95% CI: 2.1 - 18.4)more likely to develop cervical cancer than those in the 21-30 years' age category. The odds of getting cancer were higher in women living with HIV. Women with HIV were more than twice (OR = 2.3, 95% CI: 1.1 - 4.9) as likely to have cervical cancer as those who were HIV negative (Table 3).

For peer review only

Table 3: Association between cancer, age and HIV status

		Cancer (%)	Non cancer (%)	Unadjusted odds ratio (95% CI)†	Adjusted odds ratio (95% CI)†
Age (years)	20 and below	0 (0.0)	2 (100.0)		
	21 - 30 (reference)	7 (12.3)	50 (87.2)	-	
	31 - 40	25 (14.6)	146 (85.4)	1.2 (0.5 -3.0)	1.2 (0.5- 2.9)
	41 - 50	23 (17.7)	107 (82.3)	1.5 (0.6 - 3.8)	1.5 (0.6 - 3.8)
	51 - 60	18 (22.2)	63 (77.8)	2.0 (0.8 - 5.3)	2.2 (0.8 - 5.7)
	61 - 70	13 (44.8)	16 (55.2)	5.8 (2.0 - 17.0)	6.2 (2.1 - 18.4)
	Above 70	1 (20.0)	4 (80.0)	1.8 (0.2 - 18.3)	1.9 (0.2 - 19.9)
HIV status	Negative (reference)	26 (14.4)	155 (85.6)	-	
	Positive	15 (24.6)	46 (75.4)	1.9 (1.0 - 4.0)	2.3 (1.1 - 4.9)
	Unknown	50 (20.2)	197 (79.8)		

Fourteen patients (4 cancer cases and 10 non cancer cases) had no data on age and were excluded from this analysis

†Binary logistic regression

For peer review only

DISCUSSION

To the best of our knowledge, this is the first study conducted to determine the histopathological profile of cervical biopsies in a public tertiary hospital in Mzuzu, northern region of Malawi. This study found more benign conditions (70.4% n=352) than neoplastic malignant conditions (18.2% n=91). This is in contrast with other studies undertaken in developing countries which reported more neoplastic malignant conditions than benign conditions(8,9).

Our study found that cervicitis, an inflammatory disease comprising acute cervicitis and chronic non-specific cervicitis was the most common nonmalignant condition. It accounted for 46% of all the nonmalignant tumours and 32.4% of all cervical biopsies received in this study. This was consistent to a study conducted in Nigeria which had 37.5% cervicitis of all cervical biopsies included in that study(11). Most cases of cervicitis are often due to non-specific causes or infective agents(12). Cervicitis has been highly reported in previously studies inother countries(12,13).

In this study CIN, a premalignant lesion ranked second in nonmalignant lesions accounting for 24% of all nonmalignant lesions and 17.2% of all cervical biopsies with the prevalence of CIN I, CIN II, CIN III of 34.9%, 22.1% and 43% respectively. This finding is comparable to a study conducted in Nigeria which reported CIN constituting 15% of all cervical biopsies reviewed(13). In that study however, there was a reduction in the prevalence of CIN from low grade CIN to high grade CIN(13). In our study, the high grade CIN (CIN III) was the most common. This finding indicates that invasive cervical cancer progresses from advanced stages of precancerous lesions. This shows that there is need to create community awareness and strengthen early cervical cancer screening for Malawi to have better outcomes.

In the current study 10% (n=16) of all the cervicitis cases and 30.2% (n=26) of all the CIN biopsies had HPV. This finding suggests that there is high rate of HPV infections, the causative agent of CIN in the younger age group(13). Unfortunately, the data on population-based, age specific prevalence of HPV are not available in Malawi. However, WHO estimates that the overall HPV prevalence in Malawi is about 34%(7). HPV

For peer review only - http://bmjopen.bmj.com/site/about/guidelines.xhtml

infection and precancerous lesions are usually difficult to notice and develop into full blown cancer before women realize the need to seek medical care.

The rate of malignant lesions (tumours) in our study (18.2%) is comparable to 16.2% and 12.2% in studies conducted in Benin City(14), and in Enugu, in Nigeria(15) respectively. Studies done in South Africa, Saudi Arabia, India and United States, with malignant lesions at 2.42%, 4.95%, 5.5% and 5.0% respectively, are all at variance with the current study which explains that early cervical cancer screening helps to reduce the prevalence of cervical cancer(16–19).

Among the malignant tumours, SCC was the most common histological type of cervical cancer in our study. SCC accounted for 85.7% of malignant lesions cervical cancer and was also the most common diagnosis, at 15.6% of all cervical biopsies in this study. This is consistent with findings from other studies conducted worldwide (20–22). As Faduyile et al. observed the high rate of SCC in Malawi and Africa could reflect the low uptake of VIA and Pap smear test which are capable of identifying dysplastic conditions before transformation to malignancy(11). This shows that there is need for Malawi to have well organized cervical cancer screening and Pap smear test to reduce the prevalence of SCC(2).

In our study, adenocarcinorma and poorly differentiated carcinorma were 8.8% and 4.4% respectively of the total malignant diagnosis. The prevalence of adenocarcinoma and poorly differentiated carcinoma observed in this study is in contrast to other studies done in Nigeria where one study found 5.8% and 2.0% of the total malignant lesions diagnosis and another found 6.0% and 1.0% respectively(13,23). The high prevalence found in this study confirms a previous study findings by Chanza et al.(24). Chanza et al. reported that Malawian women delay to seek medical attention due to limited knowledge on symptoms and signs, limited financial resources, limited accessibility and unavailability of cancer screening facilities hence late diagnosis. These results show that early cervical cancer screening, increased awareness, better health care facilities, accessibility, improved histopathological confirmatory diagnosis and early treatment by surgeons, may reduce the cervical cancer burden in Malawi.

For peer review only - http://bmjopen.bmj.com/site/about/guidelines.xhtml

In the current study it has been observed that the diagnosis of cervical cancer was significantly associated with the age of the patients. The chances of developing cervical cancer increased with advancing age, with the risk becoming more prominent after age 60 years. Women aged 61-70 years were 6.2 times (OR = 6.2, 95% CI: 2.1 - 18.4) more likely to develop cervical cancer than those in the 21-30 years age category(Table 3). It is important to note that high quality screening programs are important to prevent cervical cancer among unvaccinated older women(25). None of the women in our study had received any HPV vaccine in their lifetime as the first round of the mass HPV vaccine in this country was administered in January 2019 mainly in schools, targeting 9-13 year old girls who had not yet become sexually active and the second round was given in January 2020. In Malawi, the integration of HPV vaccine programs with adequate screening programs in older women (aged 30-49 years) has the potential to reduce the burden of cervical cancer.

Malawi's HIV prevalence is one of the highest in the world, with 10.6% of adult population (aged 15-64) living with HIV(26). With such a high HIV prevalence in Malawi, there is an increased risk of AIDS-defining cancers including cervical cancer. In this study the probability of getting cancer were higher in women living with HIV. Women with HIV were more than twice (OR = 2.3, 95% CI: 1.1 - 4.9)as likely to have cervical cancer as those who were HIV negative (Table 3).This could be attributed to the reason that HIV-infected women are more likely than HIV-uninfected women to have incident and persistent HPV cervical infections(27).A twelve monthlycervical cancer screening, increased availability of dolutegravir (DTG) based antiretroviral treatment (ART) to HIV infected women now being provided in the country, routine viral load checks (at 6 months, 12 months and every 12 months since initiation) and timely switch to second line or third line regimens to ensure viral load suppression will eventually have an impact on benign lesions progressing to invasive cervical cancer(28). However, in this study almost half (52.74%, n=48) of all the women diagnosed with cervical cancer had an unknown HIV status. This calls for scaling up of HIV testing in cancer screening settings for early diagnosis and ART referral and further research is warranted on barriers among HIV-infected women to seeking cancer screening services despite already being in the health care system.

For peer review only - http://bmjopen.bmj.com/site/about/guidelines.xhtml

LIMITATIONS

This study used available programme health facility data and histopathological reports on cervical cancer. The use of health facility data has its own limitations, such as incompleteness and bias in the sense that information is obtained only from people who came to the facility and underwent biopsy, leaving out those that did not seek medical care and or were not biopsied and therefore cannot be generalized to the general population.

The other limitation of this study is that it is a single-hospital-based review and as such inadequate to draw conclusions, but it does shed some light on pathological pattern of cervical cancer in Malawi.

Finally, this is a retrospective study so we could not be able to extract details for example in cases where tumours were diagnosed by screening or symptoms, presence or absence of the patient co-morbidities. Nevertheless, the comprehensive histopathological pattern of cervical cancer demonstrated by this study provides evidence that could be used to inform policies, strategies and intervention for prevention of cancer in Malawi.

CONCLUSION

The SCC was the commonest malignant condition and cervicitis and CIN were the most common non malignant conditions in all the women studied. Since the frequency of cervical cancer is high, there is need for well detailed national policies to be put in place to increase detection of pre-invasive lesions, which in turn will decrease the frequency of cervical cancer in the country. The presence of chronic non-specific cervicitis in women of reproductive age is infective in origin with its attending sequelae. Intensifying screening programmes among women and provision of long term ART to the HIV infected may offer an opportunity for appropriate interventions to reduce morbidity, mortality and reduce complications among these women.

Declarations

i. Ethical approval and consent to participant

For peer review only

This study was approved by National Health Science Research Committee as part of the main study "Pathological profile of malignancies in northern Malawi-A retrospective study at Mzuzu Central Hospital" number 19/05/2316.Both the Mzuzu Central Hospital Research and Publication Committee and the Mzuzu Central Hospital Laboratory department consents were obtained for the study. The need for informed consent was exempted from institutional review board due to the nature of the study, which involved a retrospective analysis of routinely collected data.

ii. **Consent for publication:** Not applicable

iii. **Availability of data and materials**

The datasets used and/or analyzed during the current study are available from the corresponding author on reasonable request. Email: kasekapaul2016@gmail.com

ORCID: 0000-0002-6651-8000

iv. **Competing interests:** The authors declare that they have no competing interest

v. **Funding**

The study's data collection, analysis, interpretation of data and manuscript writing was funded by Pingtung Christian Hospital, Taiwan through Luke International Norway (LIN), Malawi, Grant Number: PS-IR-108001

vi. **Authors contributions**

PUK and FWS conceived and designed the study. AK, CC, PK, TJW, MC and BCM contributed to development of the study protocol and supervised data collection and entry. AK analyzed the data and PUK drafted the manuscript. All authors read and approved the final manuscript.

vii. **Acknowledgements**

The authors are sincerely grateful to all the data collectors and the Laboratory manager for providing the data. The Pingtung Christian Hospital through Luke International Norway (LIN) is also appreciated for financial support.

For peer review only - http://bmjopen.bmj.com/site/about/guidelines.xhtml

REFERENCES

1. Bruni L, Albero G, Serrano B et al. ICO/IARC Information Centre on HPVand Cancer (HPV Information Centre). Human Papillomavirus and Related Diseases in the World. Summary Report 17 June 2019.www.hpvcentre.net [Accessed 15 October 2019].
2. Bray F, Ferlay J, Soerjomataram I et al. Global cancer statistics 2018: GLOBOCAN estimates of incidence and mortality worldwide for 36 cancers in 185 countries. A Cancer J Clin. 2018;68(6):394–424. DOI: 10.3322/caac.21492.
3. Ferlay J, Soerjomataram I, Ervik M et al. IARC Publications Website - GLOBOCAN 2012: Estimated Cancer Incidence, Mortality and Prevalence Worldwide in 2012 v1.0 [Internet]. WHO, International Research on Cancer. 2012ISBN-13 (Database) 978-92-832-2447-1 [cited 2019 Nov 18]. Available from: https://publications.iarc.fr/Databases/Iarc-Cancerbases/GLOBOCAN-2012-Estimated-Cancer-Incidence-Mortality-And-Prevalence-Worldwide-In-2012-V1.0-2012
4. Rudd P, Gorman D, Meja S et al. Cervical cancer in southern Malawi: A prospective analysis of presentation, management and outcomes. Malawi Med J. 2017;29(2):124–9.PMID: 28955419.
5. Msyamboza KP, Ngwira B, D. The burden of selected chronic non-communicable diseases and their risk factors in malawi: Nationwide steps survey. PLoS One. 2011;6(5):611.DOI.org/10.1371/journal.pone.0020316https://doi.org/10.1371/journal.pone.0020316.
6. Msyamboza KP, Phiri T, Sichali W, Kwenda W, Kachale F. Cervical cancer screening uptake and challenges in Malawi from 2011 to 2015: Retrospective cohort study. BMC Public Health [Internet]. 2016;16(1):1–6. Available from: http://dx.doi.org/10.1186/s12889-016-3530-y
7. Malawi Ministry of Health and Population, UNFPA. Malawi National cervical cancer control Strategy 2016-2020 [Internet]. Unfpa. 2017. Available from: http://www.embase.com/search/results?subaction=viewrecord&from=export&id=L71343074%5Cnhttp://dx.doi.org/10.1158/1538-7445.AM2013-ADV01%5Cnhttp://fq5np7af6j.search.serialssolutions.com?sid=EMBASE&issn=00085472&id=doi:10.1158/1538-7445.AM2013-ADV01&atitle=N
8. Itano J, Taoka KN. Core curriculum for oncology nursing. Elsevier Saunders; 2005. 970 p.
9. Noncommunicable diseases country profiles 2018. Geneva: World Health Organization; 2018. Licence: CC BY-NC-SA 3.0 IGO.
10. National Statistical O. 2018 Malawi Population and Housing Census Main Report [Internet]. 2019. Available from: http://10.150.72.21:6510/www.nsomalawi.mw/images/stories/data_on_line/demography/census_2018/2018 Malawi Population and Housing Census Main Report.pdf
11. Omoniyi-esan OG, Osasan SA, Ojo OS. Non-neoplastic diseases of the cervix in Nigerians : A histopathological study. African Heal Sci Vol. 2006;6(2):76–80.PMCID: PMC1831974 PMID: 16916295.
12. Nwachokor FN, Forae GD. Morphological spectrum of non - neoplastic lesions of

the uterine cervix in Warri , South - South , Nigeria. 2013;16(4):429–32.DOI: 10.4103/1119-3077.116883.
13. Faduyile FA, Soyemi SS, Wright KO et al. Histopathological study of surgical cervical biopsies in Lagos ,. Trop J Obstet Gynaecol. 2017;34(2):124–8.DOI: 10.4103/TJOG.TJOG_28_17.
14. Obaseki D, Nwafor C. Cervical Cancer Screening In Benin City, South-South Nigeria [Internet]. Vol. 5, IOSR Journal of Dental and Medical Sciences (IOSR-JDMS) e-ISSN. 2013 [cited 2019 Nov 18]. e-ISSN: 2279-0853, p-ISSN: 2279-0861. www.iosrjournals.org. Available from: www.iosrjournals.org
15. Chukwuali LI, Onuigho WIB, Mgbor NC. Cervical cancer screening in Enugu, Nigeria. Trop J Obs Gynaecol. 2003;20:109–12.DOI: 10.4314/tjog.v20i2.14412.
16. Fonn S, Bloch B, Mabina M et al. Prevalence of pre-cancerous leisons and cervical cancer in South Africa-A Multicentre study. SAMJ. 2002;92(2):148–56. PMID: 11894653.
17. Balaha MH, Moghannum MS Al, Ghowinem NA et al. Cytological pattern of cervical Papanicolaou smear in eastern region of Saudi Arabia. J Cytol. 2011;28(4):173–177. DOI: 10.4103/0970-9371.86343

. 18. Patel MM, Pandya AN, Modi J. Cervical pap smear study and its utility in cancer screening, to specify the strategy for cervical cancer control. Natl J COMMUNITY Med. 2011;2(1):49–51. ISSN: 0976 3325.
19. Insinga RP, Glass AG, Rush BB. Diagnoses and outcomes in cervical cancer screening: A population-based study. Am J Obstet Gynecol. 2004 Jul;191(1):105–13. https://doi.org/10.1016/j.ajog.2004.01.043.
20. Okoye C. Histopathological pattern of cervical cancer in Benin City, Nigeria. J Med Investig Pract. 2014 Oct 1;9(4):147–147. link.gale.com/apps/doc/A414542015/AONE?u=nysl_ca_ind&sid=googleScholar&xid=7c182fa6. Accessed 18 Aug. 2021. Gale Document Number: GALE|A414542015.

21. Pathak T, Pun C, Shrestha S et al. Incidence trends and histopathological pattern of cervical malignancies at BP koirala memorial centre hospital, Nepal. J Pathol Nepal. 2013;3:386–9. DOI: https://doi.org/10.3126/jpn.v3i5.7863.
22. Badar F, Anwar N, Meerza F et al. Cervical Carcinoma in a Muslim Community. Asian Pacific J Cancer Prev. 2007;8:24–6. PMID: 17477766.
23. Olu-Eddo AN, Ekanem VJ, Umannah I et al. A 20 year histopathological study of cancer of the cervix in Nigerians. Nig Q J Hosp Med. 2011 Apr;21(2):149–53. PMID: 21913514.
24. Chadza E, Chirwa E, Maluwa A et al. Factors that contribute to delay in seeking cervical cancer diagnosis and treatment among women in Malawi. 2012;4(11):1015–22. DOI: 10.4236/health.2012.411155.
25. Soares GR, Vieira R da R, Pellizzer EP et al. Indications for the HPV vaccine in adolescents: A review of the literature. J Infect Public Health [Internet]. . 2015;8(2):105–16. Available from: http://dx.doi.org/10.1016/j.jiph.2014.08.011. http://dx.doi.org/10.1016/j.jiph.2014.08.011.
26. Malawi Population-based HIV Impact Assessment Survey - MPHIA 2015 - 2016

For peer review only - http://bmjopen.bmj.com/site/about/guidelines.xhtml

[Internet]. Available from: https://phia.icap.columbia.edu/wp-content/uploads/2019/08/MPHIA-Final-Report_web.pdf

27. Abraham AG, Strickler HD, Jing Y et al. Invasive cervical cancer risk among HIV-infected women: A North American multi-cohort collaboration prospective study. J Acquir Immune Defic Syndr. 2013;62(4):405–13. doi: 10.1097/QAI.0b013e31828177d7.

28. Ministry of Health and P. Malawi Guidelines for Clinical Management of HIV in Children and Adults (Third Edition, 2016) [Internet]. Lilongwe; 2016 [cited 2019 Nov 19]. Available from: www.hiv.health.gov.mw

For peer review only

Figure 1

Figure 1 is a flow diagram showing the process involved in coming up with a histopathological diagnosis at KCH/UNC laboratory in Lilongwe.

For peer review only

For peer review only - http://bmjopen.bmj.com/site/about/guidelines.xhtml

Figure 1. Process involved in specimen processing at KCH/UNC laboratory

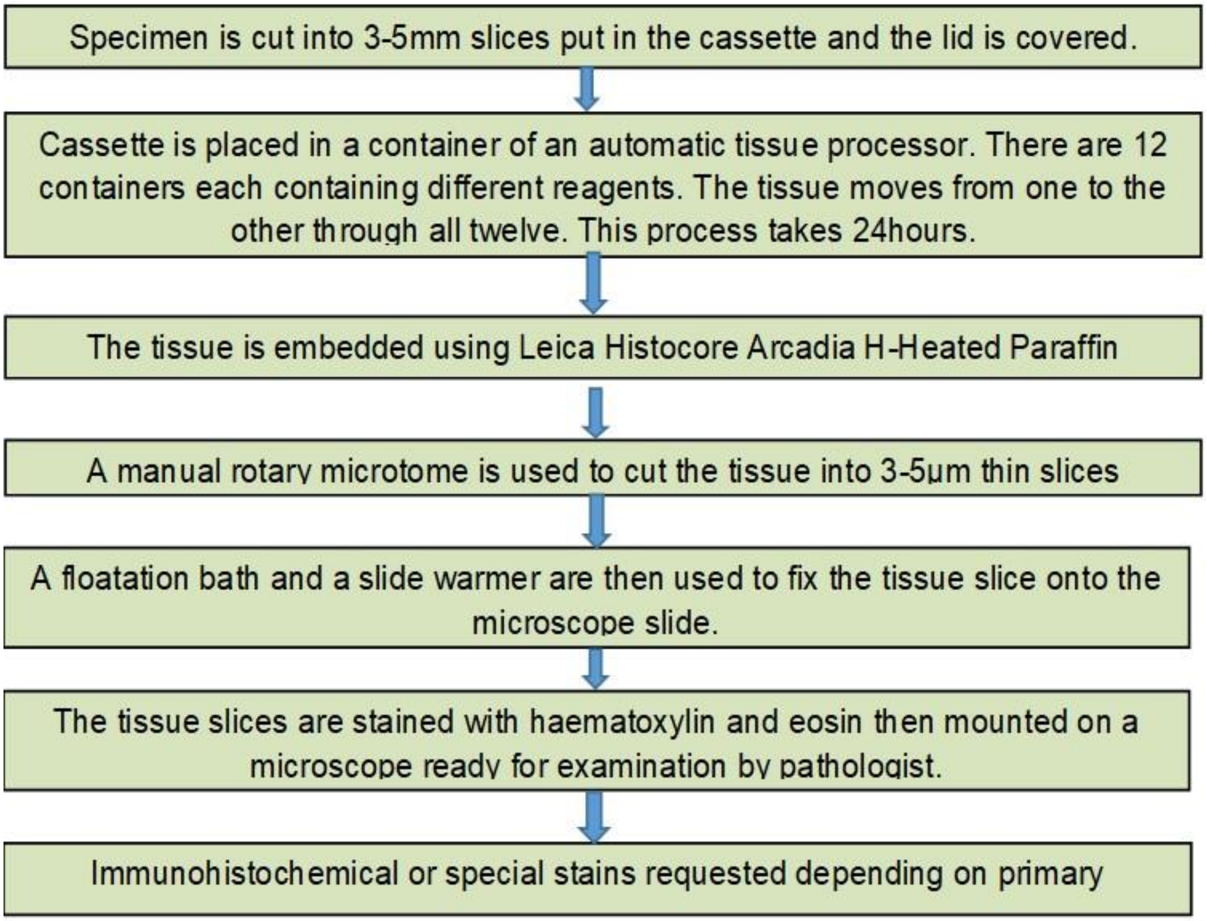

only

STROBE 2007 (v4) Statement—Checklist of items that should be included in reports of *cross-sectional studies*

Section/Topic	Item #	Recommendation	Reported on page #
**Title and abstract**	1	(*a*) Indicate the study's design with a commonly used term in the title or the abstract	1
		(*b*) Provide in the abstract an informative and balanced summary of what was done and what was found	1
**Introduction**			
Background/rationale	2	Explain the scientific background and rationale for the investigation being reported	3
Objectives	3	State specific objectives, including any prespecified hypotheses	4
**Methods**			
Study design	4	Present key elements of study design early in the paper	4
Setting	5	Describe the setting, locations, and relevant dates, including periods of recruitment, exposure, follow-up, and data collection	4
Participants	6	(*a*) Give the eligibility criteria, and the sources and methods of selection of participants	5
Variables	7	Clearly define all outcomes, exposures, predictors, potential confounders, and effect modifiers. Give diagnostic criteria, if applicable	5
Data sources/ measurement	8*	For each variable of interest, give sources of data and details of methods of assessment (measurement). Describe comparability of assessment methods if there is more than one group	5
Bias	9	Describe any efforts to address potential sources of bias	*5*
Study size	10	Explain how the study size was arrived at	5
Quantitative variables	11	Explain how quantitative variables were handled in the analyses. If applicable, describe which groupings were chosen and why	5
Statistical methods	12	(*a*) Describe all statistical methods, including those used to control for confounding	5
		(*b*) Describe any methods used to examine subgroups and interactions	5
		(*c*) Explain how missing data were addressed	5
		(*d*) If applicable, describe analytical methods taking account of sampling strategy	5
		(*e*) Describe any sensitivity analyses	5
**Results**			6

For peer review only

Participants	13*	(a) Report numbers of individuals at each stage of study—eg numbers potentially eligible, examined for eligibility, confirmed eligible, included in the study, completing follow-up, and analysed	6
		(b) Give reasons for non-participation at each stage	Not Applicable
		(c) Consider use of a flow diagram	Not Applicable
Descriptive data	14*	(a) Give characteristics of study participants (eg demographic, clinical, social) and information on exposures and potential confounders	6
		(b) Indicate number of participants with missing data for each variable of interest	6
Outcome data	15*	Report numbers of outcome events or summary measures	6
Main results	16	(*a*) Give unadjusted estimates and, if applicable, confounder-adjusted estimates and their precision (eg, 95% confidence interval). Make clear which confounders were adjusted for and why they were included	6
		(*b*) Report category boundaries when continuous variables were categorized	6
		(*c*) If relevant, consider translating estimates of relative risk into absolute risk for a meaningful time period	6
Other analyses	17	Report other analyses done—eg analyses of subgroups and interactions, and sensitivity analyses	6
**Discussion**			
Key results	18	Summarise key results with reference to study objectives	10
Limitations	19	Discuss limitations of the study, taking into account sources of potential bias or imprecision. Discuss both direction and magnitude of any potential bias	13
Interpretation	20	Give a cautious overall interpretation of results considering objectives, limitations, multiplicity of analyses, results from similar studies, and other relevant evidence	13
Generalisability	21	Discuss the generalisability (external validity) of the study results	13
**Other information**			
Funding	22	Give the source of funding and the role of the funders for the present study and, if applicable, for the original study on which the present article is based	14

*Give information separately for cases and controls in case-control studies and, if applicable, for exposed and unexposed groups in cohort and cross-sectional studies.

Note: An Explanation and Elaboration article discusses each checklist item and gives methodological background and published examples of transparent reporting. The STROBE checklist is best used in conjunction with this article (freely available on the Web sites of PLoS Medicine at http://www.plosmedicine.org/, Annals of Internal Medicine at http://www.annals.org/, and Epidemiology at http://www.epidem.com/). Information on the STROBE Initiative is available at www.strobe-statement.org.

For peer review only

Histopathological Profile of Cervical Biopsies in Northern Malawi: A Retrospective Cross-sectional Study

Paul Uchizi Kaseka[1*], Alfred Kayira[1], Chikondi Sharon Chimbatata[1], Master Chisale[1,2,3,] Pocha Kamudumuli[4], Tsung-Shu Joseph Wu[2,5], BalwaniChingatichifwe Mbakaya,[6,7]Frank Watson Sinyiza[1]

[1]Mzuzu Central Hospital, Mzuzu City, Malawi

[2]Luke International, Mzuzu City, Malawi

[3]Mzuzu University, Faculty of Sciences, Technology and Innovations, Biological Sciences, Mzuzu City, Malawi

[4]University of Maryland Global Initiative Corporation, Baltimore, Lilongwe City, Malawi

[5]Pingtung Christian Hospital, Taiwan

[6]University of Livingstonia, Department of Public Health, Malawi

[7]Mzuzu University, Faculty of Health Sciences, Mzuzu City, Malawi

*Corresponding author: kasekapaul2016@gmail.com

ABSTRACT

Objectives: According to the World Health Organization (2014), cervical cancer is the second most common cancer in women globally. More than 85% of the global cervical cancer morbidity and mortality occur in developing countries and the highest risk region is in Eastern and Southern Africa. Malawi has the highest age standardized rate of cervical cancer in the world. This study was carried out to determine the histopathological profile of cervical biopsies in a public tertiary hospital in Mzuzu, northern region of Malawi.

Setting: A public tertiary hospital in Mzuzu, northern region of Malawi

Participants: This was a retrospective study of all cervical biopsy specimen reports received in a public tertiary hospital in northern Malawi over a period of 5 years from July 2013-June 2018. Demographic, clinical and diagnostic data was obtained from original histopathology reports.

For peer review only - http://bmjopen.bmj.com/site/about/guidelines.xhtml

Results: A total of 500 cervical biopsy reports were reviewed during the study period. The mean age of the patients was 41.99±12.5. Age ranged from 15 to 80 years. Cervicitis accounted for 46.0% (n=162) of the total nonmalignant lesions seen, followed by cervical intraepithelial neoplasm (CIN), at 24.4% (n=86) and endocervical polyp, at 20.5% (n=72). Squamous cell carcinoma (SCC) accounted for 15.6% (n=78) of the total cervical biopsies studied and 85.7% of all total malignant lesions. Adenocarcinoma and undifferentiated carcinoma were 8.8% and 4.4% respectively of the total malignant diagnosis. All patients with malignant lesions had HIV.

Conclusion: Our study shows that cervicitis and squamous cell carcinoma were most common among nonmalignant and malignant cervical biopsies respectively. Since the frequency of cervical cancer is high, there is need to have well detailed national policies to be put in place to increase detection of pre-invasive lesions in order to reduce the prevalence of cervical cancer.

Key words: Cervical biopsies, cervical cancer, cervical intraepithelial neoplasm, cervicitis, malignant

Strengths and limitation of this study

- The study was able to determine the prevalence and associations of multiple exposures and outcomes.
- This being a retrospective cross-section study, the participants were neither deliberately exposed nor treated; thus, there were no ethical difficulties.
- This study depended on data that were entered into clinical database and not collected in a predesigned proforma as per specific requirements of the study as a result some records were excluded due to missing of certain crucial information.
- Since in retrospective studies, researchers have no control over the exposure of cases versus controls, these unrecognized confounders may have influenced the results.
- The study is a single-hospital-based review and as such inadequate to draw conclusions, but it does shed some light on pathological pattern of cervical cancer in Malawi.

For peer review only - http://bmjopen.bmj.com/site/about/guidelines.xhtml

BACKGROUND

Cervical cancer, after breast cancer, is the second most common cancer in women aged 15 to 44 years and it is the third leading cause of cancer in females worldwide(1). According to the International Agency for Research on Cancer(IARC) estimates, there are 570,000 new cases of cervical cancer annually, resulting into more than 311,000 deaths in 2018 globally(2). Most of the global burden lies in less developed countries, with sub-Saharan Africa (SSA) having the largest age-standardized incidence and mortality rates. Malawi has the highest cervical cancer incidence and mortality in the world with age-standardized rate (ASR) of 75.9 and 49.8 per 100,000 population respectively(3). World Health Organization (WHO) estimates suggest that every year there are at least 3,684 new cases of cervical cancer in Malawi and over 2,314 die from the disease(3).The exact number of cervical cancer morbidity and mortality among Malawian women, is not clear. This could partly be due to unrecorded or underreported cases because of the pathological based cancer registry not being maintained, and also lack of a national system of death certification(4). However, the 2010 National population-based cancer registry indicates that among females, cancer of the cervix was the commonest, accounting for 45.4% of all cases followed by Kaposi's sarcoma (21.1%), cancer of the oesophagus (8.2%), breast cancer (4.6%) and non-Hodgkin lymphoma (4.1%)(5).

The Ministry of Health and Population of the government of Malawi, through the Sexual and Reproductive Health Directorate has implemented a cervical cancer screen-and-treat programme using visual inspection with acetic acid (VIA) approach since 2004, with women between 30-50 years as the main target (6). Women, no younger than 30 years, are offered three (3) free smears, with a 10 years interval in between each smear. Those screened for first time at the age of 55 or more have only one smear if the first smear is normal(6). Cervical screening using VIA is increasingly available in local clinics through the National Cervical Cancer Control Programme(6,7). From clinics, patients are referred to District Hospitals and/or Central Hospitals. At Mzuzu Central Hospital, women with suspected cervical cancer are managed at gynaecology and/or oncology departments. Women with low-grade squamous intraepithelial lesions require

For peer review only - http://bmjopen.bmj.com/site/about/guidelines.xhtml

re-screening within a 12 months' period, whilst those with high-grade lesions are referred for colposcopy.

The standard method for diagnosis of cervical precancerous lesions is histopathological examination of tissue obtained through biopsy guided by colposcopy. When abnormalities are identified, cervical biopsy confirms the diagnosis of cancer(8).This study was carried out to determine the histopathological profile of cervical biopsies in a public tertiary hospital in Mzuzu, northern region of Malawi. The specific objectives were to determine the prevalence of both precancerous and cancerous cervical lesions; characterization of precancerous and cancerous lesions and risk factors of cervical cancer. Understanding the histological pattern of cervical cancer could guide development of focused preventive, care and treatment guidelines to address shortfalls in the care that is provided to cervical cancer patients in Malawi. Ultimately, this could contribute to the global target of 25% reduction of premature mortality from non communicable diseases (NCDs) by the year 2025(9).

MATERIALS AND METHODS

Design, setting and population

This was a retrospective study of all cervical biopsies reports received from Kamuzu Central Hospital/University of North Carolina (KCH/UNC) pathology laboratory in Lilongwe for Mzuzu Central Hospital (MCH), the only public tertiary hospital in the northern region of Malawi. This hospital does not have a functional pathology laboratory and relies on the KCH/UNC pathology laboratory for its services. The hospital is located in the northern part of Malawi catering for a population of about 2,289,780 million people(10)and serving 5 government District Hospitals, 3 Christian Health Association of Malawi (CHAM) hospitals and several private hospitals and clinics.

While some women presented to the gynaecological clinic asymptomatically through screening after VIA indicating a cancer, others presented with symptoms as referrals from a local clinic or other district hospitals from northern region. The most common symptoms were vaginal bleeding (often post coital), vaginal discharge with dysuria,

For peer review only - http://bmjopen.bmj.com/site/about/guidelines.xhtml

abdominal pain, vomiting and weight loss.

Tissue specimens were collected and preserved in 10% buffered formalin solution and then transported to Kamuzu Central Hospital/ University of North Carolina (KCH/UNC) pathology laboratory in Lilongwe. The KCH/UNC laboratory adheres to international quality assurance standards. The flow diagram (see Figure 1 attached) shows the process involved in coming up with a histopathological diagnosis at KCH/UNC laboratory in Lilongwe.

Within the 5-year period of the study (July 2013 to June 2018), a total of 2294 biopsy reports were received from KCH/UNC Histopathology laboratory. Data extracted included age, year, anatomic site, nature of specimen, clinical diagnosis, histopathological diagnosis, HIV status and whether the specimen was nonmalignant or malignant. Out of 2294 biopsy reports 500 were cervical biopsy reports representing 21.8% of the total biopsy reports received. In this study a total of 500 cervical cancer pathology reports were analyzed. Eleven reports which had missing demographic and clinical data or had inconclusive results were excluded. HIV status was defined as whether the patient was HIV sero reactive (positive), negative or not tested (unknown) when the biopsy was being taken. HPV status was defined as the patient sample being positive or negative upon histopathology examination. HPV was diagnosed histologically through observation of dysplastic changes in the superficial cervical epithelium that are consistent with HPV infection. These changes include koilocytosis and chronic inflammation. Histologically, koilocytosis is characterized by perinuclear cavitation, enlarged nucleus with coarse chromatin making it stain dark (hyperchromasia) with Lugol's iodine solution, irregular nuclear membranes and a rim of condensed cytoplasm around the perinuclear cavitation which gives the cells a 'halo' or cleared-out appearance around the dysplastic nucleus. Chronic inflammation on the other hand is characterized by infiltration of inflammatory cells (lymphocytes) into the cervical tissue.

Patient and public involvement

No patient involved

Data analysis

Data were entered in Microsoft excel 2016, validated and cleaned before importing into Stata, version 13.0 (Stata Corp. LP, College Station, TX, United States of America) for

For peer review only - http://bmjopen.bmj.com/site/about/guidelines.xhtml

analysis. Descriptive analyses were performed to summarize patients' sociodemographic and clinical characteristics. A multivariate logistic regression was used to estimate the magnitude of the association between predictor variables (age and HIV status) and cancerat 95% confidence level.

Ethical clearance

This study was approved by National Health Science Research Committee (NHSRC) as part of the main study "Pathological profile of malignancies in northern Malawi-A retrospective study at Mzuzu Central Hospital"number 19/05/2316.Both the MCH Research and Publication Committee and the MCH Laboratory department consents were obtained for the study. The need for informed consent was exempted from the institutional review board due to the nature of the study, which involved a retrospective analysis of routinely collected data.

RESULTS

Within the 5-year period of the study (July 2013 to June 2018), a total of 500 biopsy reports were received from KCH/ UNC Pathology laboratory. The mean age of patients included in this study was 41.99 ± 12.5. The age range was 15-80 years. Most of the cervical biopsies were from patients in the 31-40 age group (34.8% n=174). Twelve percent of the cervical biopsies were from HIV positive patients and 51% of the results had unknown HIV result (Table 1).

Table 1. Demographic profile of cervical biopsies

Parameter		Biopsies analysed	
		Frequency	Percentage (%)
Age(Mean)years	20 and below (17)	3	0.6
	21-30(27)	57	11.4
	31-40(36)	174	34.8

For peer review only - http://bmjopen.bmj.com/site/about/guidelines.xhtml

	41-50(45)	132	26.4
	51-60(54)	84	16.8
	61-70(65)	29	5.8
	Above 70(75)	7	1.4
	Missing	14	2.8
HIV status	Positive	61	12.2
	Negative	184	36.8
	Unknown (not documented)	255	51.0

Of all the cervical biopsies studied, 91 (18.2%) were malignant. Squamous cell carcinoma (SCC) accounted for 85.7% of all malignant lesions (Table 2). Cervicitis accounted for 46.0% (n=162) of the total nonmalignant lesions seen. Ten percent of cervicitis cases and 30.2% of CIN cases had HPV respectively (Table 2).

Table 2. Histopathological diagnosis of cervical biopsies

Parameter	Frequency	Within group percentage(%)	Percentage(%)total
**Normal cervical tissue**	**46**		**9.2**
**Nonmalignant lesions**	**352**		**70.4**
Cervicitis*	162	46.0	32.4
Cervical intraepithelial neoplasm (CIN)*	86	24.4	17.2
Endocervical polyp	72	20.5	14.4
Carcinoma in situ	4	1.1	0.8
Nabothian cyst	4	1.1	0.8
Schistosomiasis	5	1.4	1.0
Condyloma (warts)	11	3.1	2.2

For peer review only - http://bmjopen.bmj.com/site/about/guidelines.xhtml

Others	8	2.3	1.6
**Malignant**	**91**		**18.2**
Squamous cell carcinoma	78	85.7	15.6
Adenocarcinoma	8	8.8	1.6
Undifferentiated	4	4.4	0.8
Carcinosaroma	1	1.1	0.2
**Inconclusive result**	**11**		**2.2**
**Total**	**500**		**100**

*Cervicitis with HPV= 10% (16/162)   *CIN with HPV= 30.2% (26/86)

*CIN 1 = 34.9% (30/86), CIN 2 = 22.1% (19/86), CIN 3 = 43.0% (37/86)

The chances of developing cervical cancer increased with advancing age, with the risk becoming more prominent after age 60 years. Women aged 61-70 years were 6.2 times (OR = 6.2, 95% CI: 2.1 - 18.4)more likely to develop cervical cancer than those in the 21-30 years' age category. The odds of getting cancer were higher in women living with HIV. Women with HIV were more than twice (OR = 2.3, 95% CI: 1.1 - 4.9) as likely to have cervical cancer as those who were HIV negative (Table 3).

For peer review only - http://bmjopen.bmj.com/site/about/guidelines.xhtml

Table 3: Association between cancer, age and HIV status

		Cancer (%)	Non cancer (%)	Unadjusted odds ratio (95% CI)†	Adjusted odds ratio (95% CI)†
Age (years)	20 and below	0 (0.0)	2 (100.0)		
	21 - 30 (reference)	7 (12.3)	50 (87.2)	-	
	31 - 40	25 (14.6)	146 (85.4)	1.2 (0.5 -3.0)	1.2 (0.5- 2.9)
	41 - 50	23 (17.7)	107 (82.3)	1.5 (0.6 - 3.8)	1.5 (0.6 - 3.8)
	51 - 60	18 (22.2)	63 (77.8)	2.0 (0.8 - 5.3)	2.2 (0.8 - 5.7)
	61 - 70	13 (44.8)	16 (55.2)	5.8 (2.0 - 17.0)	6.2 (2.1 - 18.4)
	Above 70	1 (20.0)	4 (80.0)	1.8 (0.2 - 18.3)	1.9 (0.2 - 19.9)
HIV status	Negative (reference)	26 (14.4)	155 (85.6)	-	
	Positive	15 (24.6)	46 (75.4)	1.9 (1.0 - 4.0)	2.3 (1.1 - 4.9)
	Unknown	50 (20.2)	197 (79.8)		

Fourteen patients (4 cancer cases and 10 non cancer cases) had no data on age and were excluded from this analysis

†Binary logistic regression

For peer review only

DISCUSSION

To the best of our knowledge, this is the first study conducted to determine the histopathological profile of cervical biopsies in a public tertiary hospital in Mzuzu, northern region of Malawi. This study found more benign conditions (70.4% n=352) than neoplastic malignant conditions (18.2% n=91). This is in contrast with other studies undertaken in developing countries which reported more neoplastic malignant conditions than benign conditions(8,9).

Our study found that cervicitis, an inflammatory disease comprising acute cervicitis and chronic non-specific cervicitis was the most common nonmalignant condition. It accounted for 46% of all the nonmalignant tumours and 32.4% of all cervical biopsies received in this study. This was consistent to a study conducted in Nigeria which had 37.5% cervicitis of all cervical biopsies included in that study(11). Most cases of cervicitis are often due to non-specific causes or infective agents(12). Cervicitis has been highly reported in previously studies inother countries(12,13).

In this study CIN, a premalignant lesion ranked second in nonmalignant lesions accounting for 24% of all nonmalignant lesions and 17.2% of all cervical biopsies with the prevalence of CIN I, CIN II, CIN III of 34.9%, 22.1% and 43% respectively. This finding is comparable to a study conducted in Nigeria which reported CIN constituting 15% of all cervical biopsies reviewed(13). In that study however, there was a reduction in the prevalence of CIN from low grade CIN to high grade CIN(13). In our study, the high grade CIN (CIN III) was the most common. This finding indicates that invasive cervical cancer progresses from advanced stages of precancerous lesions. This shows that there is need to create community awareness and strengthen early cervical cancer screening for Malawi to have better outcomes.

In the current study 10% (n=16) of all the cervicitis cases and 30.2% (n=26) of all the CIN biopsies had HPV. This finding suggests that there is high rate of HPV infections, the causative agent of CIN in the younger age group(13). Unfortunately, the data on population-based, age specific prevalence of HPV are not available in Malawi. However, WHO estimates that the overall HPV prevalence in Malawi is about 34%(7). HPV

For peer review only - http://bmjopen.bmj.com/site/about/guidelines.xhtml

infection and precancerous lesions are usually difficult to notice and develop into full blown cancer before women realize the need to seek medical care.

The rate of malignant lesions (tumours) in our study (18.2%) is comparable to 16.2% and 12.2% in studies conducted in Benin City(14), and in Enugu, in Nigeria(15) respectively. Studies done in South Africa, Saudi Arabia, India and United States, with malignant lesions at 2.42%, 4.95%, 5.5% and 5.0% respectively, are all at variance with the current study which explains that early cervical cancer screening helps to reduce the prevalence of cervical cancer(16–19).

Among the malignant tumours, SCC was the most common histological type of cervical cancer in our study. SCC accounted for 85.7% of malignant lesions cervical cancer and was also the most common diagnosis, at 15.6% of all cervical biopsies in this study. This is consistent with findings from other studies conducted worldwide (20–22). As Faduyile et al. observed the high rate of SCC in Malawi and Africa could reflect the low uptake of VIA and Pap smear test which are capable of identifying dysplastic conditions before transformation to malignancy(11). This shows that there is need for Malawi to have well organized cervical cancer screening and Pap smear test to reduce the prevalence of SCC(2).

In our study, adenocarcinorma and poorly differentiated carcinorma were 8.8% and 4.4% respectively of the total malignant diagnosis. The prevalence of adenocarcinoma and poorly differentiated carcinoma observed in this study is in contrast to other studies done in Nigeria where one study found 5.8% and 2.0% of the total malignant lesions diagnosis and another found 6.0% and 1.0% respectively(13,23). The high prevalence found in this study confirms a previous study findings by Chanza et al.(24). Chanza et al. reported that Malawian women delay to seek medical attention due to limited knowledge on symptoms and signs, limited financial resources, limited accessibility and unavailability of cancer screening facilities hence late diagnosis. These results show that early cervical cancer screening, increased awareness, better health care facilities, accessibility, improved histopathological confirmatory diagnosis and early treatment by surgeons, may reduce the cervical cancer burden in Malawi.

For peer review only - http://bmjopen.bmj.com/site/about/guidelines.xhtml

In the current study it has been observed that the diagnosis of cervical cancer was significantly associated with the age of the patients. The chances of developing cervical cancer increased with advancing age, with the risk becoming more prominent after age 60 years. Women aged 61-70 years were 6.2 times (OR = 6.2, 95% CI: 2.1 - 18.4) more likely to develop cervical cancer than those in the 21-30 years age category(Table 3). It is important to note that high quality screening programs are important to prevent cervical cancer among unvaccinated older women(25). None of the women in our study had received any HPV vaccine in their lifetime as the first round of the mass HPV vaccine in this country was administered in January 2019 mainly in schools, targeting 9-13 year old girls who had not yet become sexually active and the second round was given in January 2020. In Malawi, the integration of HPV vaccine programs with adequate screening programs in older women (aged 30-49 years) has the potential to reduce the burden of cervical cancer.

Malawi's HIV prevalence is one of the highest in the world, with 10.6% of adult population (aged 15-64) living with HIV(26). With such a high HIV prevalence in Malawi, there is an increased risk of AIDS-defining cancers including cervical cancer. In this study the probability of getting cancer were higher in women living with HIV. Women with HIV were more than twice (OR = 2.3, 95% CI: 1.1 - 4.9)as likely to have cervical cancer as those who were HIV negative (Table 3).This could be attributed to the reason that HIV-infected women are more likely than HIV-uninfected women to have incident and persistent HPV cervical infections(27).A twelve monthlycervical cancer screening, increased availability of dolutegravir (DTG) based antiretroviral treatment (ART) to HIV infected women now being provided in the country, routine viral load checks (at 6 months, 12 months and every 12 months since initiation) and timely switch to second line or third line regimens to ensure viral load suppression will eventually have an impact on benign lesions progressing to invasive cervical cancer(28). However, in this study almost half (52.74%, n=48) of all the women diagnosed with cervical cancer had an unknown HIV status. This calls for scaling up of HIV testing in cancer screening settings for early diagnosis and ART referral and further research is warranted on barriers among HIV-infected women to seeking cancer screening services despite already being in the health care system.

For peer review only - http://bmjopen.bmj.com/site/about/guidelines.xhtml

LIMITATIONS

This study used available programme health facility data and histopathological reports on cervical cancer. The use of health facility data has its own limitations, such as incompleteness and bias in the sense that information is obtained only from people who came to the facility and underwent biopsy, leaving out those that did not seek medical care and or were not biopsied and therefore cannot be generalized to the general population.

The other limitation of this study is that it is a single-hospital-based review and as such inadequate to draw conclusions, but it does shed some light on pathological pattern of cervical cancer in Malawi.

Finally, this is a retrospective study so we could not be able to extract details for example in cases where tumours were diagnosed by screening or symptoms, presence or absence of the patient co-morbidities. Nevertheless, the comprehensive histopathological pattern of cervical cancer demonstrated by this study provides evidence that could be used to inform policies, strategies and intervention for prevention of cancer in Malawi.

CONCLUSION

The SCC was the commonest malignant condition and cervicitis and CIN were the most common non malignant conditions in all the women studied. Since the frequency of cervical cancer is high, there is need for well detailed national policies to be put in place to increase detection of pre-invasive lesions, which in turn will decrease the frequency of cervical cancer in the country. The presence of chronic non-specific cervicitis in women of reproductive age is infective in origin with its attending sequelae. Intensifying screening programmes among women and provision of long term ART to the HIV infected may offer an opportunity for appropriate interventions to reduce morbidity, mortality and reduce complications among these women.

Declarations

i. Ethical approval and consent to participant

For peer review only - http://bmjopen.bmj.com/site/about/guidelines.xhtml

This study was approved by National Health Science Research Committee as part of the main study “Pathological profile of malignancies in northern Malawi-A retrospective study at Mzuzu Central Hospital” number 19/05/2316.Both the Mzuzu Central Hospital Research and Publication Committee and the Mzuzu Central Hospital Laboratory department consents were obtained for the study. The need for informed consent was exempted from institutional review board due to the nature of the study, which involved a retrospective analysis of routinely collected data.

ii. **Consent for publication:** Not applicable

iii. **Availability of data and materials**

The datasets used and/or analyzed during the current study are available from the corresponding author on reasonable request. Email: kasekapaul2016@gmail.com

ORCID: 0000-0002-6651-8000

iv. **Competing interests:** The authors declare that they have no competing interest

v. **Funding**

The study’s data collection, analysis, interpretation of data and manuscript writing was funded by Pingtung Christian Hospital, Taiwan through Luke International Norway (LIN), Malawi, Grant Number: PS-IR-108001

vi. **Authors contributions**

PUK and FWS conceived and designed the study. AK, CC, PK, TJW, MC and BCM contributed to development of the study protocol and supervised data collection and entry. AK analyzed the data and PUK drafted the manuscript. All authors read and approved the final manuscript.

vii. **Acknowledgements**

The authors are sincerely grateful to all the data collectors and the Laboratory manager for providing the data. The Pingtung Christian Hospital through Luke International Norway (LIN) is also appreciated for financial support.

For peer review only - http://bmjopen.bmj.com/site/about/guidelines.xhtml

REFERENCES

1. Bruni L, Albero G, Serrano B et al. ICO/IARC Information Centre on HPVand Cancer (HPV Information Centre). Human Papillomavirus and Related Diseases in the World. Summary Report 17 June 2019.www.hpvcentre.net [Accessed 15 October 2019].
2. Bray F, Ferlay J, Soerjomataram I et al. Global cancer statistics 2018: GLOBOCAN estimates of incidence and mortality worldwide for 36 cancers in 185 countries. A Cancer J Clin. 2018;68(6):394–424. DOI: 10.3322/caac.21492.
3. Ferlay J, Soerjomataram I, Ervik M et al. IARC Publications Website - GLOBOCAN 2012: Estimated Cancer Incidence, Mortality and Prevalence Worldwide in 2012 v1.0 [Internet]. WHO, International Research on Cancer. 2012ISBN-13 (Database) 978-92-832-2447-1 [cited 2019 Nov 18]. Available from: https://publications.iarc.fr/Databases/Iarc-Cancerbases/GLOBOCAN-2012-Estimated-Cancer-Incidence-Mortality-And-Prevalence-Worldwide-In-2012-V1.0-2012
4. Rudd P, Gorman D, Meja S et al. Cervical cancer in southern Malawi: A prospective analysis of presentation, management and outcomes. Malawi Med J. 2017;29(2):124–9.PMID: 28955419.
5. Msyamboza KP, Ngwira B, D. The burden of selected chronic non-communicable diseases and their risk factors in malawi: Nationwide steps survey. PLoS One. 2011;6(5):611.DOI.org/10.1371/journal.pone.0020316https://doi.org/10.1371/journal.pone.0020316.
6. Msyamboza KP, Phiri T, Sichali W, Kwenda W, Kachale F. Cervical cancer screening uptake and challenges in Malawi from 2011 to 2015: Retrospective cohort study. BMC Public Health [Internet]. 2016;16(1):1–6. Available from: http://dx.doi.org/10.1186/s12889-016-3530-y
7. Malawi Ministry of Health and Population, UNFPA. Malawi National cervical cancer control Strategy 2016-2020 [Internet]. Unfpa. 2017. Available from: http://www.embase.com/search/results?subaction=viewrecord&from=export&id=L71343074%5Cnhttp://dx.doi.org/10.1158/1538-7445.AM2013-ADV01%5Cnhttp://fq5np7af6j.search.serialssolutions.com?sid=EMBASE&issn=00085472&id=doi:10.1158/1538-7445.AM2013-ADV01&atitle=N
8. Itano J, Taoka KN. Core curriculum for oncology nursing. Elsevier Saunders; 2005. 970 p.
9. Noncommunicable diseases country profiles 2018. Geneva: World Health Organization; 2018. Licence: CC BY-NC-SA 3.0 IGO.
10. National Statistical O. 2018 Malawi Population and Housing Census Main Report [Internet]. 2019. Available from: http://10.150.72.21:6510/www.nsomalawi.mw/images/stories/data_on_line/demography/census_2018/2018 Malawi Population and Housing Census Main Report.pdf
11. Omoniyi-esan OG, Osasan SA, Ojo OS. Non-neoplastic diseases of the cervix in Nigerians : A histopathological study. African Heal Sci Vol. 2006;6(2):76–80.PMCID: PMC1831974 PMID: 16916295.
12. Nwachokor FN, Forae GD. Morphological spectrum of non - neoplastic lesions of

For peer review only - http://bmjopen.bmj.com/site/about/guidelines.xhtml

the uterine cervix in Warri , South ‐ South , Nigeria. 2013;16(4):429–32.DOI: 10.4103/1119-3077.116883.
13. Faduyile FA, Soyemi SS, Wright KO et al. Histopathological study of surgical cervical biopsies in Lagos ,. Trop J Obstet Gynaecol. 2017;34(2):124–8.DOI: 10.4103/TJOG.TJOG_28_17.
14. Obaseki D, Nwafor C. Cervical Cancer Screening In Benin City, South-South Nigeria [Internet]. Vol. 5, IOSR Journal of Dental and Medical Sciences (IOSR-JDMS) e-ISSN. 2013 [cited 2019 Nov 18]. e-ISSN: 2279-0853, p-ISSN: 2279-0861. www.iosrjournals.org. Available from: www.iosrjournals.org
15. Chukwuali LI, Onuigho WIB, Mgbor NC. Cervical cancer screening in Enugu, Nigeria. Trop J Obs Gynaecol. 2003;20:109–12.DOI: 10.4314/tjog.v20i2.14412.
16. Fonn S, Bloch B, Mabina M et al. Prevalence of pre-cancerous leisons and cervical cancer in South Africa-A Multicentre study. SAMJ. 2002;92(2):148–56. PMID: 11894653.
17. Balaha MH, Moghannum MS Al, Ghowinem NA et al. Cytological pattern of cervical Papanicolaou smear in eastern region of Saudi Arabia. J Cytol. 2011;28(4):173–177. DOI: 10.4103/0970-9371.86343

. 18. Patel MM, Pandya AN, Modi J. Cervical pap smear study and its utility in cancer screening, to specify the strategy for cervical cancer control. Natl J COMMUNITY Med. 2011;2(1):49–51. ISSN: 0976 3325.
19. Insinga RP, Glass AG, Rush BB. Diagnoses and outcomes in cervical cancer screening: A population-based study. Am J Obstet Gynecol. 2004 Jul;191(1):105–13. https://doi.org/10.1016/j.ajog.2004.01.043.
20. Okoye C. Histopathological pattern of cervical cancer in Benin City, Nigeria. J Med Investig Pract. 2014 Oct 1;9(4):147–147. link.gale.com/apps/doc/A414542015/AONE?u=nysl_ca_ind&sid=googleScholar&xid=7c182fa6. Accessed 18 Aug. 2021. Gale Document Number: GALE|A414542015.

21. Pathak T, Pun C, Shrestha S et al. Incidence trends and histopathological pattern of cervical malignancies at BP koirala memorial centre hospital, Nepal. J Pathol Nepal. 2013;3:386–9. DOI: https://doi.org/10.3126/jpn.v3i5.7863.
22. Badar F, Anwar N, Meerza F et al. Cervical Carcinoma in a Muslim Community. Asian Pacific J Cancer Prev. 2007;8:24–6. PMID: 17477766.
23. Olu-Eddo AN, Ekanem VJ, Umannah I et al. A 20 year histopathological study of cancer of the cervix in Nigerians. Nig Q J Hosp Med. 2011 Apr;21(2):149–53. PMID: 21913514.
24. Chadza E, Chirwa E, Maluwa A et al. Factors that contribute to delay in seeking cervical cancer diagnosis and treatment among women in Malawi. 2012;4(11):1015–22. DOI: 10.4236/health.2012.411155.
25. Soares GR, Vieira R da R, Pellizzer EP et al. Indications for the HPV vaccine in adolescents: A review of the literature. J Infect Public Health [Internet]. . 2015;8(2):105–16. Available from: http://dx.doi.org/10.1016/j.jiph.2014.08.011. http://dx.doi.org/10.1016/j.jiph.2014.08.011.
26. Malawi Population-based HIV Impact Assessment Survey - MPHIA 2015 - 2016

For peer review only - http://bmjopen.bmj.com/site/about/guidelines.xhtml

[Internet]. Available from: https://phia.icap.columbia.edu/wp-content/uploads/2019/08/MPHIA-Final-Report_web.pdf
27. Abraham AG, Strickler HD, Jing Y et al. Invasive cervical cancer risk among HIV-infected women: A North American multi-cohort collaboration prospective study. J Acquir Immune Defic Syndr. 2013;62(4):405–13. doi: 10.1097/QAI.0b013e31828177d7.
28. Ministry of Health and P. Malawi Guidelines for Clinical Management of HIV in Children and Adults (Third Edition, 2016) [Internet]. Lilongwe; 2016 [cited 2019 Nov 19]. Available from: www.hiv.health.gov.mw

For peer review only

Figure 1

Figure 1 is a flow diagram showing the process involved in coming up with a histopathological diagnosis at KCH/UNC laboratory in Lilongwe.

For peer review only

For peer review only - http://bmjopen.bmj.com/site/about/guidelines.xhtml
